# Discovery and optimisation studies of antimalarial phenotypic hits

**DOI:** 10.1016/j.ejmech.2015.08.044

**Published:** 2015-10-20

**Authors:** Alka Mital, Dinakaran Murugesan, Marcel Kaiser, Clive Yeates, Ian H. Gilbert

**Affiliations:** a Division of Biological Chemistry and Drug Discovery, College of Life Sciences, University of Dundee, Sir James Black Centre, Dundee DD1 5EH, UK; b Swiss Tropical and Public Health Institute, Postfach, Socinstrasse 57, 4002 Basel, Switzerland; c University Basel, Petersplatz 1, 4003 Basel, Switzerland; d InPharma Consultancy, Herts, UK

**Keywords:** *Plasmodium falciparum*, Malaria, Medicinal chemistry, Phenotypic hit, DMPK, drug metabolism and pharmacokinetics, SAR, structure-activity relationship, WHO, World Health Organisation, WHO-TDR, World Health Organisation Programme for Research and Training in Tropical Diseases

## Abstract

There is an urgent need for the development of new antimalarial compounds. As a result of a phenotypic screen, several compounds with potent activity against the parasite *Plasmodium falciparum* were identified. Characterization of these compounds is discussed, along with approaches to optimise the physicochemical properties. The *in vitro* antimalarial activity of these compounds against *P. falciparum* K1 had EC_50_ values in the range of 0.09–29 μM, and generally good selectivity (typically >100-fold) compared to a mammalian cell line (L6). One example showed no significant activity against a rodent model of malaria, and more work is needed to optimise these compounds.

## Highlights

• Malaria is a leading cause of morbidity and mortality globally
• This paper reports optimisation of hits active against *Plasmodium falciparum*.
• Approaches to improve physicochemical and pharmacokinetic properties.
• Compounds profiled in multiple assays.

## Graphical abstract

**Figure:**
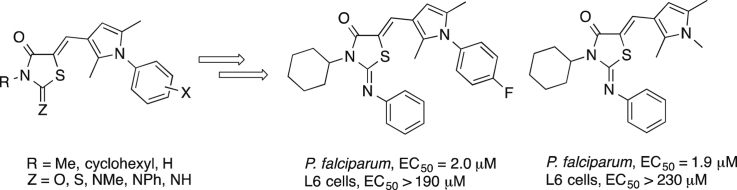

## 1 Introduction

Malaria is a serious endemic disease and is a major threat to public health in more than 100 countries [1], [2]. It affects about 200 million people per year, with approximately 580,000 associated deaths [3], [4]. In addition malaria exerts a huge economic toll in endemic countries [3]. The need for a continual supply of new antimalarial therapeutics is still as relevant as ever.

Malaria is caused by protozoan parasites of the species *Plasmodium* [5], with *Plasmodium falciparum* being responsible for most malaria-related deaths. In many areas malaria parasites have developed resistance to chemotherapeutic agents such as chloroquine, mefloquine, and sulfadoxine/pyrimethamine. Therefore, an urgent need exists to develop new classes of antimalarial drugs that operate by novel mechanisms of action.

**Fig. 1:**
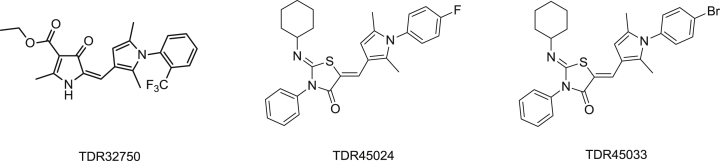
The hit TDR32750 and follow-up compounds.

We have recently reported the identification of a hit (**TDR32750**) [6], [7] from a screen of the ChemDiv5000 ‘maximally structurally diverse’ compound collection against *P. falciparum*. This screen was carried out by the World Health Organisation Programme for Research and Training in Tropical Medicine (Fig. 1). **TDR32750** showed potent activity against *P. falciparum* (EC_50_ = 9 nM), and good selectivity compared to L6 mammalian cells (>2000-fold). In order to follow up on the hit, other analogues from ChemDiv and PrincetonBio were screened. This led to identification of two more hits, **TDR45024** and **TDR45033** (Fig. 1), which shared the *N*-arylpyrrole found in **TDR32750**.

**Fig. 2:**
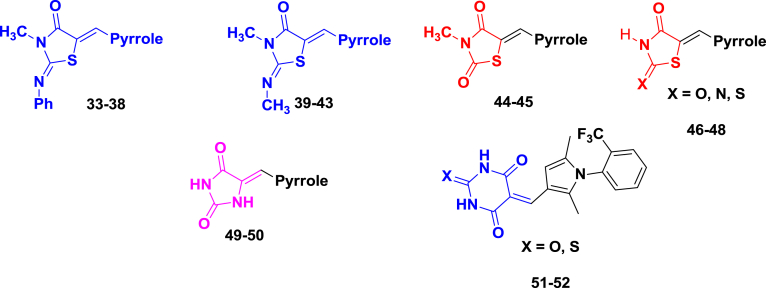
Outline of derivatives prepared.

In this paper we report the follow up of these compounds, **TDR45024** and **TDR45033**; systematic structure-activity relationship studies were undertaken with the aim of improving anti-parasitic activity, and to generate compounds with drug-like physicochemical and pharmacokinetic properties. The studies encompassed variation of the phenyl ring attached to the pyrrole, modification of the pyrrole and modification of the thiazolidinedione ring. The activity of compounds against the chloroquine and pyrimethamine resistant (K1) strain of *P. falciparum* is reported, as well as a counter-screen (EC_50_) against the L6 murine cell line, to provide an indication of selectivity (Table 1, Fig. 2).

**Table 1:**
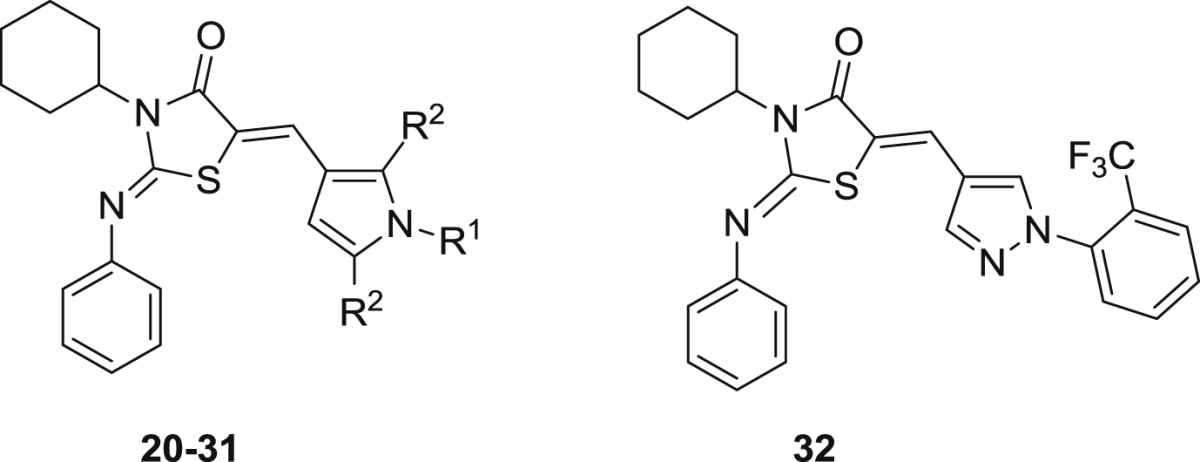
*In vitro* activity of phenyliminothiazolidinones against *P. falciparum* and L6 cells.

Compound *(Z,Z-isomers)*	R^1^	R^2^	*P. falc*.^a^ EC_50_(μM)	L-6 cells^b^ EC_50_(μM)	cLogP	cLogD pH 7.4
**20**		Me	2.0	>190	6.6	2.7
**21**		Me	0.42	>170	7.2	3.1
**22**		Me	0.25	>200	6.6	2.6
**23**		Me	0.25	>170	7.5	3.2
**24**		Me	1.9	>170	7.5	3.2
**25**		Me	0.78	>170	7.5	3.2
**26**		Me	0.09	>170	6.5	3.1
**27**		Me	0.61	71	5.4	1.9
**28**		H	2.3	>180	6.7	2.9
**29**		H	1.6	>200	6.1	2.2
**30**		Me	3.4	23	5.1	1.5
**31**		Me	1.9	>230	5.6	1.5
**32**	–	–	1.9	>180	6.1	2.6

a *Plasmodium falciparum*.

b Measure of cytotoxicity; nd: not determined. Controls: for *P. falciparum* K1, chloroquine, EC_50_ = 0.1 μg/ml; for cytotoxicity (L6 cells), podophyllotoxin, EC_50_ = 0.005 μg/ml. The EC_50_ values are the data are means of two independent assays run in singleton. Yields for compounds **20**–**32** are 40–80%.

## 2 Results and discussion

### 2.1 Synthesis of cyclohexyl-2-(phenylimino)-4-thiazolidinedione analogues (**20**–**32**)

**Scheme 1:**
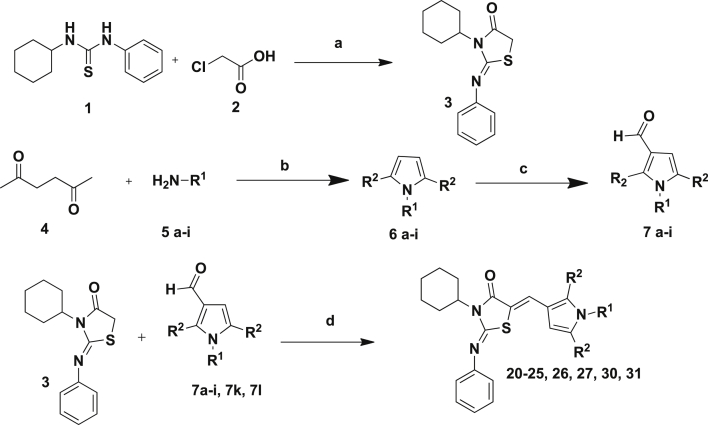
General Synthetic Approach to thiazolidin-4-ones: (a) sodium acetate, ethanol, 0 °C, 30 min, 20%; (b) p-toluenesulfonic acid bound with silica gel, microwave (0–400 W at 2.45 GHz), 180 °C, 15–20 min, 80–90%; or p-toluenesulfonic acid, toluene, 90 °C, 3 h, Dean–Stark Apparatus; (c) phosphorous oxychloride, DMF, 100 °C, 3 h, 80–95%; (d) piperidine, ethanol, 3 h, reflux, 20%.

The thiazolidinedione core [8], [9] (**3**) was prepared by condensation of the commercially available 1-cyclohexyl-3-phenyl-2-thiourea (**1**) with monochloroacetic acid (**2**; Scheme 1). This was then condensed with 3-formylpyrroles to yield the desired products (**20**–**32**). We have previously reported the preparation of a number of the 3-formylpyrroles (**7**) used here [7]. They were obtained by condensation of the appropriate aniline with 2,5-hexanedione (**4**) (Paal–Knorr pyrrole synthesis), and subsequent Vilsmeier–Haack formylation.

**Scheme 2:**
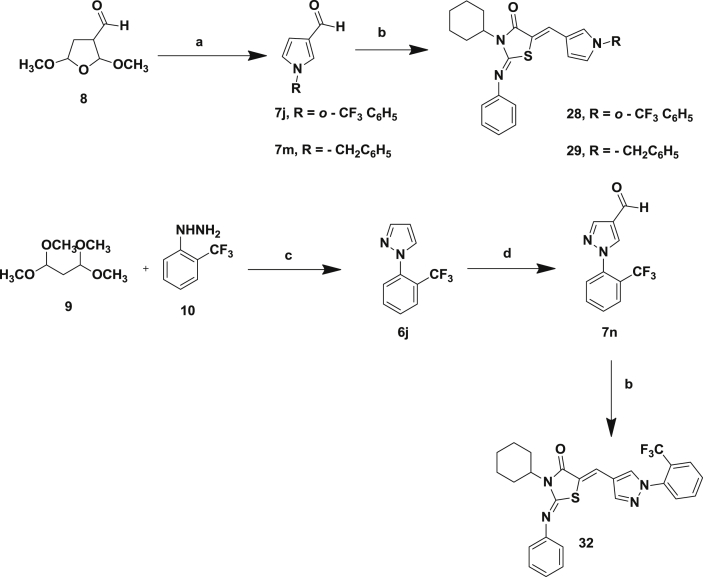
(a) 2-Trifluoromethyl aniline (**7j**) or benzyl amine (**7m**), glacial acetic acid, 90 °C, 80%; (b) thiazolidinedione (**3**), piperidine, ethanol, 3 h, reflux, 80–95%; (c) ethanol, H_2_O, HCl, 90 °C, 3 h; (d) phosphorous oxychloride, DMF, 100 °C, 3 h, 80–95% yield.

3-Formyl pyrroles without the 2,5-dimethyl substitution were prepared (Scheme 2). We have previously reported the preparation of compound **7j** through condensation of *o*-trifluoromethyl aniline with 2,5-dimethoxy tetrahydrofuran-3-carboxaldehyde (**8**) [7]. Compound **7m** was similarly prepared using benzylamine. The pyrroles **7j** [10], [11] and **7m** were then condensed with the thiazolidinedione core to give the desired products **28** and **29**. Similarly the pyrrole could be replaced by a pyrazole [12]. As we have previously reported, the 3-formylpyrazole **7n** was prepared by condensation of 1,1,3,3-tetramethoxypropane **9** and 2-(trifluoromethyl)phenyl hydrazine **10**, followed by Vilsmeier–Haack formylation [7]. This was condensed with the thiazolidinedione core **3** to give the desired product **32** in a yield of 65%.

### 2.2 Modification of the thiazolidinedione core

**Scheme 3:**
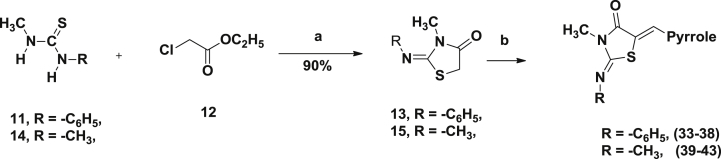
(a) Anhydrous sodium acetate, ethanol; (b) 3-formyl pyrrole (**7c**–**7n**), piperdine, ethanol, 3 Å molecular sieves, 90 °C, 10 h.

The lipophilic cyclohexyl and N-phenyl groups from the thiazolidinedione core were replaced with methyls (Scheme 3), in order to improve the physicochemical properties of the molecule.

**Scheme 4:**
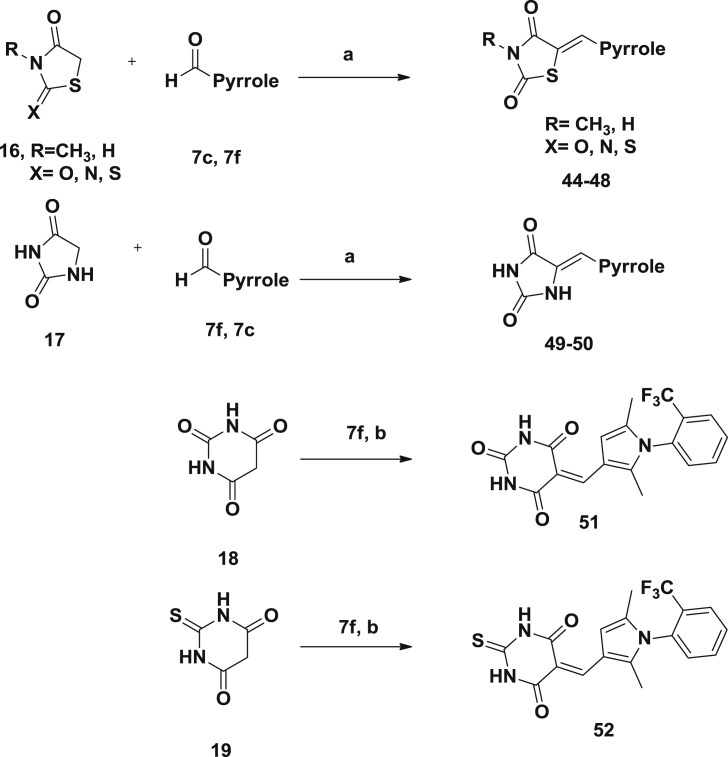
(a) Piperdine, ethanol, 3 Å molecular sieves, 90 °C, 8 h; (b) conc HCl, r.t, 1 h, 70–95%.

We also investigated replacing the N-imino group on the thiazolidinedione with an oxygen and replacing the thiazolidinedione ring altogether with a barbituric acid moiety (Scheme 4).

### 2.3 Stereochemistry

There are two potential stereocenters; in compounds **20**–**42**, the exocyclic double bond to the imine; the exocyclic double bond to the pyrrole. Literature precedent [13], suggests the stereochemistry of these double bonds is (*Z*,*Z*). In the case of the exocyclic double bond to the imine, an E-configuration is highly unlikely as there would be steric clashes between the substituent on the imine (in the case of compound **20**, the phenyl), with the substituent on the ring nitrogen (in the case of compound **20**, the cyclohexyl). In the case of the exocyclic double bond to the pyrrole, the ^1^H NMR signal of the exocyclic alkenic proton is indicative of the Z-stereoisomer. As in the literature precedent [13], this proton (HC=C) is de-shielded by the carbonyl group and for most of the compounds (**20**–**43**) appeared with chemical shifts in the range of 7.65–8.35 ppm.

## 3 Biology. *In vitro* activity

The compounds **(20**–**32**) were assayed against *P. falciparum* K1 strain [14], and counter-screened in mammalian L6-cells [15].

### 3.1 Modifications of the cyclohexyl-2-(phenylimino)-4-thiazolidinedione derivatives (**20**–**32**, Table 1)

The synthesized compounds were found to show a fairly flat range of activities against *P. falciparum* K1 (EC_50_'s 0.09–3.4 μM), with the R^1^ group having relatively little affect on activity.

• The original hit molecules **20** and **21** were re-synthesised, and identity and purity confirmed; they had EC_50_ of 2.0 μM and 0.42 μM respectively.
• The other variants around the phenyl ring showed similar activities: the unsubstituted phenyl (**22**, EC_50_ = 0.25 μM); the trifluoromethyl substituents (**23**, EC_50_ = 0.25 μM, **24**, EC_50_ = 1.9 μM, **25**, EC_50_ = 0.78 μM). The only exception to this is the morpholine substituted derivative which showed a slight improvement in potency (**26**, EC_50_ = 0.09 μM).
• Replacement of the phenyl ring with a morpholine (**27**, EC_50_ = 0.61 μM), a hydrogen (**30**, EC_50_ = 3.4 μM) or a methyl (**31**, EC_50_ = 1.9 μM) did not significantly affect activity.
• Changes to the pyrrole ring also had little effect on potency: including removal of the methyl groups (**28**, EC_50_ = 2.3 μM); or changing the pyrrole to a pyrazole (**32**, EC_50_ = 1.9 μM).

### 3.2 Modifications to the cyclohexyl-2-(phenylimino)-4-thiazolidinedione core (**33**–**52**; Table 2)

• Replacing the cyclohexyl ring on the thiazolidinedione with methyl (**33**–**38**) gave a drop in potency of between 2 and 10-fold (EC_50_ = 0.99–4.6 μM). However, given the drop in molecular weight and logP, this is a relatively small loss in activity, but gives an improvement in physicochemical properties.

**Table 2:** *In vitro* activity against antiplasmodial and cytotoxic activity.

Compound^c^	Structure	P. falc^a^ EC_50_(μM)	L-6 cells^b^ EC_50_(μM)	cLogP	CLogD pH 7.4
**33**		2.6	>230	4.5	1.8
**34**		2.2	>200	5.7	2.6
**35**		1.5	120	4.5	2.4
**36**		0.99	>210	3.3	1.1
**37**		3.9	>240	4.0	1.3
**38**		4.6	180	4.3	1.9
**39**		>13	>220	3.2	1.2
**40**		5.6	78	2.0	−0.14
**41**		4.7	4	3.7	1.1
**42**		9.5	>290	2.8	0.10
**43**		3.6	34	3.1	0.7
**44**		1.9	64	4.0	4.0
**45**		2.2	>290	3.4	3.4
**46**		8.4	43	4.0	4.0
**47**		5.8	56	4.0	1.8
**48**		21	9	4.2	4.2
**49**		13	84	3.4	3.4
**50**		11	>320	2.3	2.3
**51**		16	120	2.9	2.9
**52**		29	16	3.3	3.3

a *Plasmodium falciparum*.

b Measure of cytotoxicity; nd: not determined. Controls: for *P. falciparum*, chloroquine K1, EC_50_ = 0.1 μg/ml; for cytotoxicity (L6 cells), podophyllotoxin, EC_50_ = 0.005 μg/ml. The EC_50_ values are the data are means of two independent assays run in singleton. Yields for compounds **33**–**52**, 40–80%.

c Configuration as (*Z,Z*) 33–44, as (*Z*) 45,49–50, as (*E*) 46–48.
• Replacement of the N-phenyl imine with an N-methyl imine (**39**–**43**) also led to a small drop in activity (EC_50_ = 3.6->13 μM), compared to the parent analogues.
• Replacement of the imine with an oxygen **44**–**46**, **49**, **50** with or without a methyl group on the thiazolidinedione nitrogen (**45**) gave a similar level in activity (EC_50_ = 1.9–13 μM).
• The imino (**47**) (EC_50_ = 5.8 μM) and thio (**48**) (EC_50_ = 21 μM) analogues had low activity.
• The barbituric acid analogues were relatively inactive (**51**, **52**) (EC_50_ = 16 and 29 μM, respectively).
• In addition, some of these compounds showed reduced selectivity based in the L-6 assay.

## 4 Pharmacology

### 4.1 *In vivo* efficacy studies in *Plasmodium berghei* mouse model

To establish proof of concept, compound **20** was taken forward to the *P. berghei* mouse model [16]. Compound **20** as a suspension in aqueous DMSO was dosed *intraperitoneally* at 50 mg/kg for 4 days but resulted in no significant reduction in parasitaemia or increase in survival time (Table 3).

**Table 3:** *In vivo* antimalarial Activity against *P. berghei* (ANKA).

	Dose (mg/kg/day × 4)	Route	% Reduction parasitaemia *P. berghei* ANKA GFP	Survival (days)
**20**	50	*ip*^a^	12	6
Chloroquine	10	*ip*	99.97	20
Control				7

a ip: intraperitoneal route of administration.

## 5 Physiochemical properties and *in vitro* DMPK

The physicochemical properties of **20** were evaluated using a combination of *in silico* and experimental techniques, and the metabolic stability was assessed *in vitro* using human and mouse liver microsomes, (Table 4). **Compound 20** meets the Lipinski criteria, except for the high lipophilicity with a logD of 7.1, which explains the poor aqueous solubility at pH 2 and 6.5.

**Table 4:** Physicochemical data of **20**.

Compound	MW	H-bond		PSA	FRB	LogD^a^ pH 7.4	Solubility (μg/ml)	
		Don	Acc				pH 2.0	pH 6.5
**20**	473.6	0	4	63	4	7.1	<0.8	0.8–1.6

a Value measured using the chromatographic gLogD technique.

Compound **20** underwent rapid NADPH-dependent degradation upon incubation with human and mouse liver microsomes, (Table 5). There was no species difference in the relative rate of metabolism between human and mouse liver microsomes, and no obvious metabolites were detected using the analytical conditions employed for the parent compound. The rapid microsomal clearance and low solubility probably explains the lack of activity of compound **20** in the mouse model.

**Table 5:** Metabolic stability of compound **20** based on NADPH-dependent degradation profiles in human/mouse liver microsomes.

Compound	Species	*In vitro* *CL*_*int*_^a^ (ml/min/mg Protein)	Microsome predicted *in vivo* CL_*int*_^a^ (mL/min/kg)	Microsome predicted hepatic extraction ratio E_H_	Metabolites detected
20	Human	0.36	42	0.67	None
	Mouse	0.055	216	0.71	None

a Intrinsic clearance.

## 6 Conclusion

As a result of a phenotypic screen, some antimalarial compounds were identified. Despite activity against *P. falciparum in vitro*, an example compound was not active in a rodent model of disease, most likely due to a combination of high metabolic turnover and low aqueous solubility arising from their relatively high lipophilicity. To address these issues a number of analogues were prepared with lower lipophilicity, measured by cLogP, but unfortunately these showed no significant improvement in potency against the parasite. In order to progress this series further, compounds need to be identified with improved potency and reduced lipophilicity. A further impetus would come from an identification of the molecular target(s) of these compounds.

## 7 Materials and methods

### 7.1 General experimental information

Chemicals and solvents were purchased from Aldrich Chemical Co. or Fluka, and were used as received unless otherwise stated. Air- and moisture-sensitive reactions were carried out under an inert atmosphere of argon in oven-dried glassware. Analytical thin-layer chromatography (TLC) was performed on pre-coated TLC plates (layer 0.20 mm silica gel 60 with fluorescent indicator UV254, from Merck). Developed plates were air-dried and analysed under a UV lamp (UV 254/365 nm). Flash column chromatography was performed using pre-packed silica gel cartridges (230–400 mesh, 40–63 *μ*m, from SiliCycle) using a Teledyne ISCO Combiflash Companion or Combiflash Retrieve. Microwave irradiation was conducted using a BIOTAGE^®^ INITIATOR unit. The machine consists of a continuous focused microwave power delivery system with operator-selectable power output (0–400 W at 2.45 GHz).^1^H NMR and ^13^C NMR spectra were recorded on a Bruker Avance II 500 spectrometer (^1^H at 500.1 MHz, ^13^C at 125.8 MHz) or a Bruker DPX300 spectrometer (^1^H at 300.1 MHz). Chemical shifts (*δ*) are expressed in ppm recorded using the residual solvent as the internal reference in all cases. Signal splitting patterns are described as singlet (s), doublet (d), triplet (t), quartet (q), pentet (p), multiplet (m), broad (br), or a combination thereof. Coupling constants (*J*) are quoted to the nearest 0.1 Hz. LC-MS analyses were performed with either an Agilent HPLC 1100 series connected to a Bruker DaltonicsMicroTOF or an Agilent Technologies 1200 series HPLC connected to an Agilent Technologies 6130 quadrupole spectrometer, where both instruments were connected to an Agilent diode array detector. LC-MS chromatographic separations were conducted with a Waters Xbridge C18 column, 50 mm × 2.1 mm, 3.5 *μ*m particle size; mobile phase, water/acetonitrile +0.1% HCOOH, or water/acetonitrile + 0.1% NH_3_; linear gradient from 80:20 to 5:95 over 3.5 min and then held for 1.5 min; flow rate of 0.5 mL min^−1^. All assay compounds had a measured purity of ≥95% (by total ion current (TIC) and UV) as determined using this analytical LC−MS system. High resolution electrospray measurements were performed on a Bruker DaltonicsMicrOTOF mass spectrometer.

### 7.2 (Z)-3-Cyclohexyl-2-(phenylimino)thiazolidin-4-one (**3**)

**Figure:**
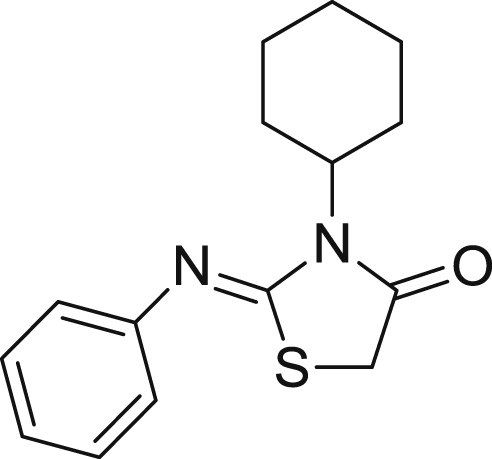

A mixture of 3-cyclohexyl-1-phenyl-2-thiourea (5 g, 21 mmol), monochloroacetic acid (2.02 g, 21 mmol), and anhydrous sodium acetate (1.75 g, 21 mmol) in absolute ethanol (100 mL) was refluxed for 8–10 h. The reaction mixture was filtered and the solvent was evaporated under reduced pressure. The residue was purified by column chromatography on a silica gel column (dichloromethane/hexane) to give (1.44 g, 25%) of **3** as a yellowish solid, mp. 88-90 °C. ^1^H NMR (500 MHz; CDCl_3_/Me_4_Si): *δ* 7.28 (m, 2H), 7.06 (m, 1H), 6.86 (m, 2H), 4.39 (m, 1H), 3.65 (s, 2H), 2.34 (m, 2H), 1.79 (d, 2H, *J* = 13.45 Hz), 1.64 (d, 2H, *J* = 4.9 Hz), 1.59 (d, 1H, *J* = 12.75 Hz), 1.30 (m, 2H), 1.17 (m,1H); ^13^C NMR (125 MHz, CDCl_3_/Me_4_Si): *δ* 172.1, 154.6, 148.5, 129.3 (2C), 124.5, 120.9 (2C), 56.1, 32.5 (2C), 28.1 (2C), 26.1, 25.1; HRMS (*m/z*): [MH^+^] calcd for C_15_H_18_FN_2_OS, 275.0154; found 275.2415.

### 7.3 General procedure for the microwave-accelerated synthesis of 2,5-dimethyl-1-aryl-1H-pyrroles (6a–i): [7]

2,5-Hexandione (**4**) (1 mmol), the appropriate aniline (**5a-i**) (1.2 equiv) and *p*-toluenesulfonic acid bound with silica gel (0.4 equiv) were mixed in oven dried pressure vials with magnetic stir bars. The vessel was placed in a microwave oven and heated (180 °C, 15–20 min) under microwave irradiation (0–400 W at 2.45 GHz), then stirred for 15 min at room temperature. The silica residue was removed by filtration, washing with DCM (10 mL) The solvent was removed under reduced pressure to give the 2,5-dimethyl-1-aryl-1*H*-pyrroles (**6a-i**) (Purity >95% by HPLC, 80–90% yield), which were used without further purification.

### 7.4 General procedure for the synthesis of 2,5-dimethyl-1-aryl-3-formylpyrroles (**7a**–**i**): [7]

As we have previously described [7] phosphorous oxychloride (6 mmol) was added dropwise to stirred ice-cooled DMF (12 mL) under a N_2_ atmosphere. The mixture was kept at room temperature for 15 min and then a solution of the appropriate 2,5-dimethyl-1-aryl-1*H*-pyrrole (**6a**–**i**) (1 mmol) in DMF (5 mL) was added and the mixture then heated at 100 °C for 3 h under a N_2_ atmosphere. After cooling 30% NaOH was added dropwise to adjust to pH ∼10. The solid precipitate was filtered, washed with water and dried in vacuo to afford the 2,5-dimethyl-1-aryl-3-formylpyrrole (**7a**–**i**) (80–95% yield), which was used without further purification.

### 7.5 General condensation procedure for cyclohexyl-2-(phenylimino)thiazolidin-4-one; 3-methyl-2-(phenylimino)thiazolidine-4-one; 3-methyl-2-(methylimino)thiazolidine-4-one; thiazolidine-2,4-dione; 3-methyl thiazolidine-2,4-dione and imidazolidine-2,4-dione derivatives (**20**–**54**)

To a solution of cyclohexyl-2-(phenylimino)-4-thiazolidineione, 3-methyl-2-(phenylimino) thiazolidine-4-one, thiazolidine-2,4-dione, 3-methyl thiazolidine-2,4-dione or imidazolidine-2,4-dione (1.0 equiv) in absolute ethanol (15 mL) was added the appropriate 2,5-dimethyl-1-aryl -3-formylpyrrole (1.1 equiv), piperidine (1.2 equiv) and 3 Ǻ molecular sieves. The mixture was heated to reflux at 90 °C for 8–10 h, cooled, filtered and concentrated in vacuo. The residue was purified by column chromatography on a silica gel column (ethylacetate/hexane or dichloromethane/hexane) and in some cases recrystallized from methanol to give **20**–**54** as generally yellowish solids.

#### 7.5.1 (2Z, 5Z)-3-Cyclohexyl-5-((1-(4-fluorophenyl)-2,5-dimethyl-1H-pyrrol-3-yl)methylene)-2-(phenylimino)thiazolidin-4-one (**20**)

**Figure:**
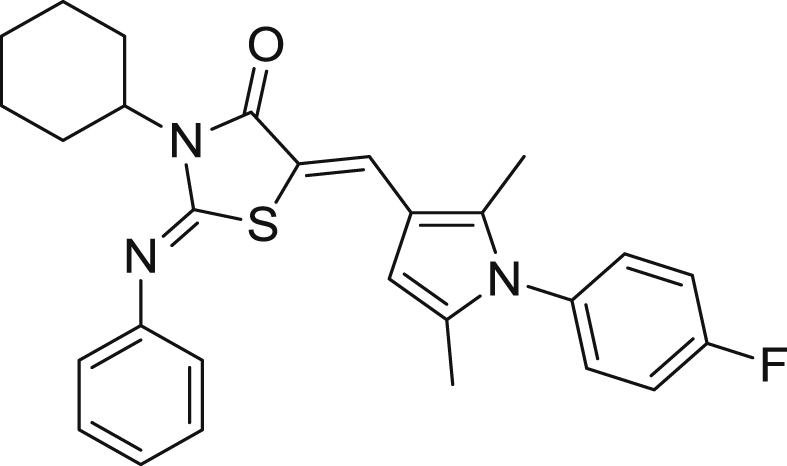

Yellowish solid, (0.100 g, 13%), mp. 225–227 °C. ^1^H NMR (500 MHz, CDCl_3_/Me_4_Si): *δ* 7.69 (s, 1H, CH=C), 7.42 (t, 2H, *J* = 7.88), 7.19 (m, 5H), 7.06 (m, 2H), 6.11 (s, 1H), 4.63 (m, 1H), 2.54 (m, 2H), 2.14 (s, 3H), 1.98 (s, 3H), 1.90 (d, 2H, *J* = 18.10), 1.81 (d, 2H, *J* = 11.45 Hz), 1.71 (d, 1H, *J* = 18.10 Hz), 1.44 (m, 2H), 1.31 (m, 1H). ^13^C NMR (125 MHz, CDCl_3_/Me_4_Si): *δ* 167.7 (C=O), 163.3, 161.4, 151.5 (C=N), 149.1(C=C), 134.5, 133.7, 131.2, 129.8, 129.7, 129.3 (2C), 124.3, 124.2, 121.4, 116.6, 116.4, 115.8, 114.5, 105.5, 55.6, 28.5 (2C), 26.2, 25.3 (2C), 12.7, 11.0. Anal. Calc for C_28_H_28_FN_3_OS (473.60): C, 71.01; H, 5.96; N, 8.87; found: C, 68.91; H, 5.64; N, 8.38. HRMS (*m/z*): [MH^+^] calcd for C_28_H_29_FN_3_OS, 474.2015; found 474.2004.

#### 7.5.2 (2Z,5Z)-3-Cyclohexyl-5-((1-(4-bromophenyl)-2,5-dimethyl-1H-pyrrol-3-yl)methylene)-2-(phenylimino)thiazolidin-4-one (**21**)

**Figure:**
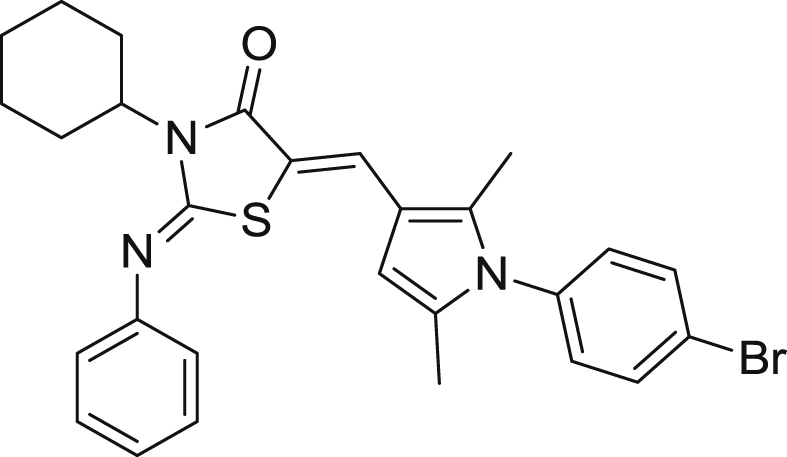

Yellowish solid, (0.470 g, 24%), mp. 243–244 °C. ^1^H NMR (500 MHz, CDCl_3_/Me_4_Si): *δ* 7.59 (s, 1H, CH=C), 7.55 (m, 2H), 7.32 (t, 2H, *J* = 7.88 Hz), 7.10 (t, 1H, *J* = 7.43 Hz), 6.97 (m, 4H), 6.02 (s, 1H), 4.53 (tt, 1H, *J* = 3.87, 4.89 Hz), 2.45 (m, 2H), 2.05 (s, 3H), 1.89 (s, 3H), 1.81 (m, 2H), 1.72 (m, 2H), 1.61 (m, 1H), 1.35 (m, 2H), 1.20 (m, 1H);^13^C NMR (125 MHz, CDCl_3_/Me_4_Si): *δ* 167.7 (C=O), 151.6 (C=N), 149.8 (C=C), 137.7, 136.8, 134.5, 134.2, 132.7, 131.3, 131.0, 129.6, 129.3, 124.3, 124.1, 122.7, 121.4, 116.0, 114.7, 112.2, 105.7, 55.6, 28.5 (2C), 26.2, 25.3 (2C), 12.7, 11.1; Anal. Calcd for C_28_H_28_BrN_3_OS (534.51): C, 62.92; H, 5.28; N, 7.86; found: C, 62.74; H, 5.19; N, 7.65. HRMS (*m/z*): [MH^+^] calcd for C_28_H_29_BrN_3_OS, 534.1215; found 534.1223.

## Appendix A Supporting information

The following is the supporting information related to this article:

Supporting information available: including synthetic routes for the synthesis of intermediates, methods for physicochemical evaluation of the compounds and the data generated, methods for the drug metabolism, pharmacokinetics, *in vitro* and *in vivo* parasite testing; chemistry experimental for compounds not included in the main text.
