# Supplementary material for: Discovery and optimisation studies of antimalarial phenotypic hits
Source: Eur J Med Chem. 2015 Oct 20;103:530–8. doi: 10.1016/j.ejmech.2015.08.044 (PMC4607724; doi:10.1016/j.ejmech.2015.08.044)
Supplement: Supplementary file 1 [file mmc1.pdf]

Supporting Information:

Discovery and Optimisation Studies of Antimalarial Phenotypic Hits

Alka Mital,[†] Dinakaran Murugesan,[†] Marcel Kaiser,[#] Clive Yeates,[∞] Ian H. Gilbert.[†*]

[†]Division of Biological Chemistry and Drug Discovery, College of Life Sciences, University of Dundee, Sir James Black Centre, Dundee, DD1 5EH, UK

[#]Swiss Tropical and Public Health Institute, Postfach, Socinstrasse 57, 4002 Basel, Switzerland

[‡]University Basel, Petersplatz 1, 4003 Basel, Switzerland

[∞]InPharma Consultancy, Herts, UK

*Author for correspondence: i.h.gilbert@dundee.ac.uk; Tel: +44 1382 386 240

Supporting Information

Table of contents

I.	General information	Page 2
II.	Chemical Preparation	Page 3
III.	Representative Spectra	Page 27
IV.	Physicochemical Evaluation	Page 39
V.	*In vitro* Metabolism	Page 39
VI.	Design and Results of the *in vitro* & *in vivo* Studies	Page 40
VII.	Additional References	Page 42

I. General information

^{1}H NMR and ^{13}C NMR spectra were recorded on a Bruker Avance II 500 spectrometer (^{1}H at 500.1 MHz, ^{13}C at 125.8 MHz) or a Bruker DPX300 spectrometer (1H at 300.1 MHz). Chemical shifts (δ) are expressed in ppm recorded using the residual solvent as the internal reference in all cases. Signal splitting patterns are described as singlet (s), doublet (d), triplet (t), quartet (q), pentet (p), multiplet (m), broad (br), or a combination thereof. Coupling constants (J) are quoted to the nearest 0.1 Hz. LC−MS analyses were performed with either an Agilent HPLC 1100 series connected to a Bruker Daltonics MicrOTOF or an Agilent Technologies 1200 series HPLC connected to an Agilent Technologies 6130 quadrupole spectrometer, where both instruments were connected to an Agilent diode array detector. LC−MS chromatographic separations were conducted with a Waters X-bridge C18 column, 50 mm × 2.1 mm, 3.5 μm particle size; mobile phase, water/acetonitrile + 0.1% HCOOH, or water/acetonitrile + 0.1% NH_3; linear gradient from 80:20 to 5:95 over 3.5 min and then held for 1.5 min; flow rate of 0.5 mL min^{-1}. All assay compounds had a measured purity of ≥95% (by TIC and UV) as determined using this analytical LC−MS system. High resolution electrospray measurements were performed on a Bruker Daltonics MicrOTOF mass spectrometer.

II. Chemical Preparation:

(Z)-3-Methyl-2-(phenylimino)thiazolidine-4-one (13).[1]

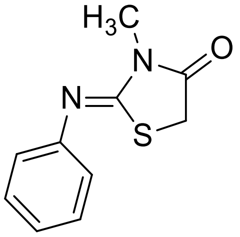

A mixture of 3-methyl-1-phenyl-2-thiourea **11** (5.28 g, 0.032 mol), ethylchloroacetate **12** (3.90 g, 0.032 mol), and anhydrous sodium acetate (5.21 g, 0.060mol) in absolute ethanol (50 mL) was refluxed for 10–12 hr. The reaction mixture was filtered and excess of ethanol was evaporated under reduced pressure. The crude product was purified by column chromatography on a silica gel column (ethylacetate/hexane) to give 5.90 g (90%) of yellowish solid product. ^{1}H NMR (500 MHz, $CDCl_3/Me_4Si$): δ 7.38 (m, 2H), 7.19 (m, 1H), 6.99 (m, 2H), 3.85 (s, 2H, -CH_2), 3.35 (s, 3H, N-CH_3). ^{13}C NMR (125 MHz, $CDCl_3/Me_4Si$): δ 171.8, 155.0, 148.0, 129.3 (2C), 124.7, 121.0 (2C, arom, N-CH_3), 32.8, 29.7. MS (ESI$^+$): (*m/z*) [MH$^+$] 207.06, 100 %.

(Z)-3-Methyl-2-(methylimino)thiazolidine-4-one (15).[1]

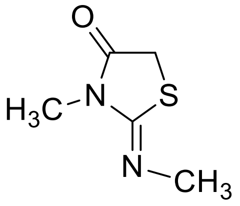

A mixture of N,N'-dimethylthiourea **14** (5.0 g, 0.048 mol), ethylchloroacetate **12** (5.88 g, 0.048 mol), and anhydrous sodium acetate (7.88 g, 0.096mol) in absolute ethanol (50 mL) was refluxed for 12 h. The reaction mixture was filtered and the solvent was evaporated under reduced pressure. The residue was purified by column chromatography on a silica gel column (ethylacetate/hexane) to give 5.20 g (75%) of yellowish solid product, mp. 69–70 ºC.^{1}H NMR (500 MHz, $CDCl_3/Me_4Si$): δ 3.84 (s, 2H, CH_2), 3.18 (dd, 6H, N-CH_3, J = 0.60, 3.18 Hz). ^{13}C NMR (125 MHz, $CDCl_3/Me_4Si$): δ 171.6 (C=O), 154.3 (C=N-), 38.5, 32.7, 29.3. MS (ESI$^+$): (*m/z*) [MH$^+$] 145.05, 100 %.

A. General procedure for the microwave-accelerated synthesis of 2,5-dimethyl-1-aryl-1*H*-pyrroles (6a-i).

Following our previously reported procedure,[2] 2,5-hexandione (**4**) (1 mmol), the appropriate aniline **5a-i** (1.2 equiv) and *p*-toluenesulfonic acid bound with silica gel (0.4 equiv) were mixed in an oven dried pressure vial with a magnetic stirrer bar. The vessel was placed in a microwave oven and heated (180 ºC, 15–20 min) under microwave irradiation (0–400 W at 2.45 GHz). After stirring for a further 15 min at room temperature the reaction mixture was filtered and the residual silica washed with DCM (10 mL). The solvent was removed under reduced pressure to give the corresponding 2,5-dimethyl-1-aryl-1*H*-pyrrole **6a–i** (80–90% yield, purity >95% by LCMS).

Compounds 6a, 6b, 6d, 6e, 6f, 6g, 6h, & 6j[3] have been already reported[2]

1-(4-Fluorophenyl)-2,5-dimethyl-1*H*-pyrrole (6a).

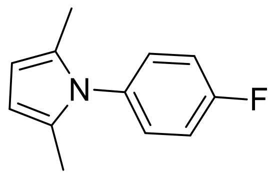

1-(4-Bromophenyl)-2,5-dimethyl-1*H*-pyrrole (6b).

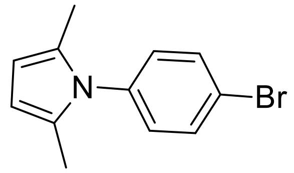

2,5-Dimethyl-1-(4-(trifluoromethyl)phenyl)-1*H*-pyrrole (6d).

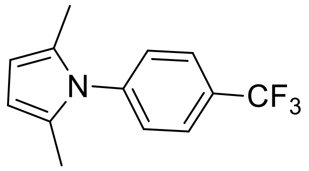

2,5-Dimethyl-1-(3-(trifluoromethyl)phenyl)-1*H*-pyrrole (6e).

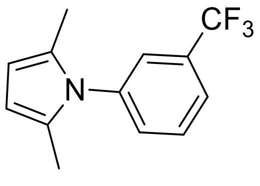

2,5-Dimethyl-1-(2-(trifluoromethyl)phenyl)-1*H*-pyrrole (6f).

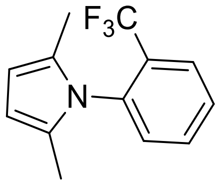

4-(4-(2,5-Dimethyl-1H-pyrrol-1-yl)phenyl)morpholine (6g).

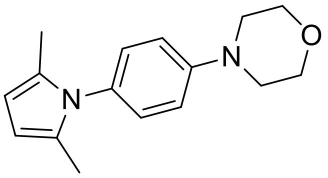

4-(2-(2,5-Dimethyl-1H-pyrrol-1-yl)ethyl)morpholine (6h).

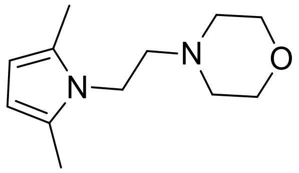

2,5-Dimethyl-1-phenyl-1*H*-pyrrole (6c).

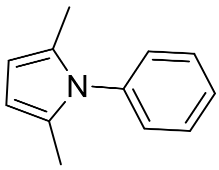

Brown solid (16.53 g, 85%), mp. 46–48 ºC (Lit. 45–46 ºC). ^{1}H NMR (500 MHz, $CDCl_3$/Me_4Si): δ 7.39 (m, 2H), 7.32 (m, 1H), 7.15 (m, 2H), 5.84 (s, 2H), 1.96 (s, 6H). ^{13}C NMR (125 MHz, $CDCl_3$/Me_4Si): δ 139.0, 129.1 (2C), 128.9, 128.3 (2C), 127.7 (2C), 105.6 (2C), 13.1 (2C). MS (ESI^{+}): (*m*/*z*)[MH^{+}]172.20, 100%.

1-(2-(2,5-Dimethyl-1H-pyrrol-1-yl)ethyl)piperazine (6i).

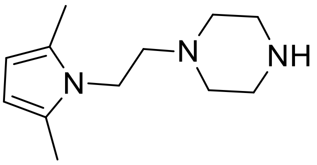

Brown liquid (1.81 g, 56.4%). (Lit. bp. 178–180 ºC; mp. 12 ºC). ^{1}H NMR (500 MHz, $CDCl_3$/Me_4Si): δ 5.79 (s, 2H), 3.91 (m, 2H), 2.93 (t, 4H, *J*= 4.85 Hz), 2.56-2.51 (m, 6H), 2.26 (s, 6H). ^{13}C NMR (125 MHz; $CDCl_3$/Me_4Si): δ 127.5 (2C), 105.4, 105.2, 59.1, 55.1 (2C), 46.1 (2C), 41.2, 12.5 (2C). MS (ESI^{+}): (*m/z*) [MH^{+}]208.18, 100 %.

1-(2-(Trifluoromethyl)phenyl)-1*H*-pyazole (6j).

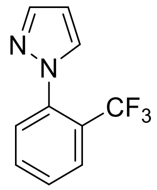

B. General Procedure for the synthesis of formylpyrroles (7a–i).

Following our previously reported procedure,[2, 4] phosphorous oxychloride (0.5 mL, 6 mmol) was added dropwise to stirred ice-cooled DMF (12 mL) under a N_2 atmosphere. The mixture was then allowed to warm to room temperature over 15 min, a solution of the pyrrole **(6a–i)** (1 mmol) in DMF (5 mL) added and the mixture heated at 100 ºC for 3 h under a N_2 atmosphere. After cooling, 30% aq NaOH was added dropwise to pH 10.0 and the resulting precipitate was filtered, washed several times with water and dried *in vacuo* to give the corresponding formyl derivative **7a–i** (80–95%), which was generally used without further purification.

Compounds 7a, 7b, 7d, 7e, 7f, 7g, 7h, 7j[5, 6] & 7n have been already reported[2]

1-(4-Fluorophenyl)-2, 5-dimethyl-1*H*-pyrrole-3-carbaldehyde (7a).

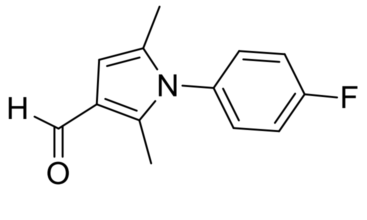

1-(4-Bromophenyl)-2, 5-dimethyl-1*H*-pyrrole-3-carbaldehyde (7b).

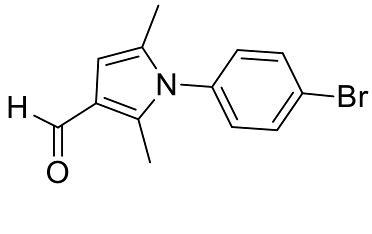

2,5-Dimethyl-1-(4-(trifluoromethyl)phenyl)-1*H*-pyrrole-3-carbaldehyde (7d).

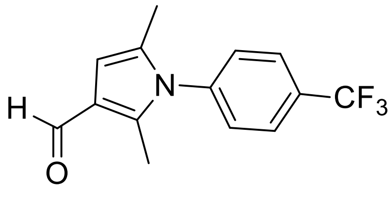

2,5-Dimethyl-1-(3-(trifluoromethyl)phenyl)-1*H*-pyrrole-3-carbaldehyde (7e).

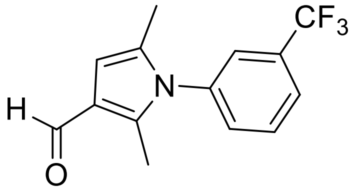

2,5-Dimethyl-1-(2-(trifluoromethyl)phenyl)-1*H*-pyrrole-3-carbaldehyde (7f).

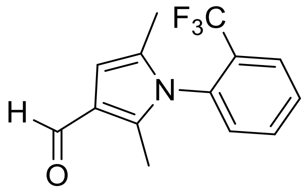

2,5-Dimethyl-1-(4-morpholinophenyl)-1*H*-pyrrole-3-carbaldehyde (7g).

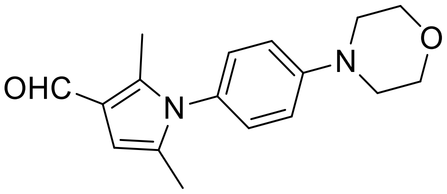

2,5-Dimethyl-1-(2-morpholinoethyl)-1*H*-pyrrole-3-carbaldehyde (7h).

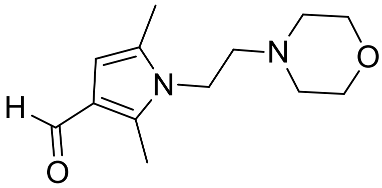

1-(2-(trifluoromethyl)phenyl)-1H-pyrrole-3-carbaldehyde (7j)

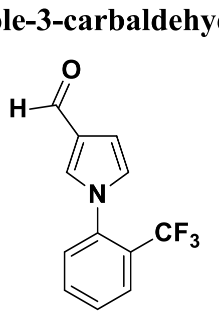

1-(2-(Trifluoromethyl)phenyl)-1*H*-pyazole-4-carbaldehyde (7n).

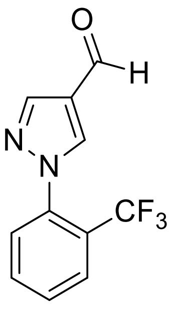

2,5-Dimethyl-1-phenyl-1*H*-pyrrole-3-carbaldehyde (7c).

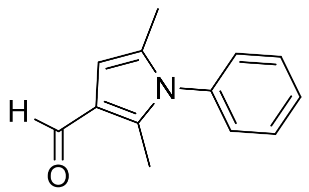

Dark brown solid (2.64 g, 90%). mp. 88–90 ºC (Lit. 89–91 ºC). ^{1}H NMR (500 MHz; $CDCl_3/Me_4Si$): δ 9.81 (s, 1H), 7.46 (m, 3H), 7.14 (m, 2H), 6.32 (s, 1H), 2.21 (s, 3H), 1.92 (s, 3H). ^{13}C NMR (125 MHz; $CDCl_3/Me_4Si$): δ 185.3, 150.3, 135.8, 135.2, 130.1, 130.0, 129.8, 127.7, 127.4, 112.0, 106.3, 12.8, 12.6. MS (ESI^{+}): (*m/z*)[MH^{+}]200.11, 100%.

2,5-Dimethyl-1-(2-(piperazin-1-yl)ethyl)-1*H*-pyrrole-3-carbaldehyde (7i).

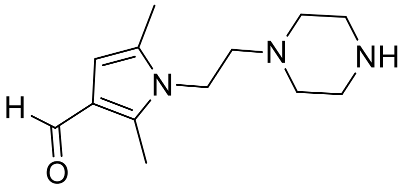

Dark brown liquid (1.25 g, 60 %). ^{1}H NMR (500 MHz; $CDCl_3/Me_4Si$): *δ* 9.67 (s, 1H), 6.15 (s, 1H), 5.63 (m, 2H), 3.80 (m, 2H), 3.44 (t, 2H, *J*= 4.78 Hz), 3.25 (t, 2H, *J*= 5.05 Hz), 2.47 (m, 2H), 2.40 (s, 3H), 2.34 (m, 2H), 2.11 (m, 3H). ^{13}C NMR (125 MHz; $CDCl_3/Me_4Si$): *δ* 185.1, 129.6, 127.4, 106.5, 105.5, 57.8, 54.2, 45.5, 43.5, 41.6, 39.9, 12.4, 10.6. MS (ESI^{+}): (*m/z*)[MH^{+}]236.17, 100%.

2,5-Dimethyl-1H-pyrrole-3-carbaldehyde (7k).

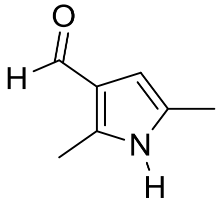

To an ice-cooled solution of 2,5-dimethylpyrrole (4 g, 0.042 mol) in dichloroethane (100 mL), was added DMF (3.84 g, 0.053 mol) at 0 ºC, followed by dropwise addition of phosphorus oxychloride (7.74 g, 0.050 mol) at 0 ºC. The reaction mixture was allowed to stir at 0 ºC for 15 min and then heated at 80-85ºC for 30 min. The mixture was cooled to room temperature and then poured into ice water, and made alkaline (pH 10–11) with aqueous NaOH (20% w/v). The aqueous phase was extracted with of dichloromethane (3 x 50 mL), and the combined organic phase was washed with water (2 x 100 mL), dried ($MgSO_4$) and concentrated *in vacuo* to yield a light brown solid (4.17 g, 80%), mp.133–135 ºC. ^{1}H NMR (500 MHz, $CDCl_3/Me_4Si$): δ 9.73 (s, 1H), 6.14 (s, 1H), 2.43 (s, 3H), 2.15 (s, 3H). ^{13}C NMR (125 MHz, $CDCl_3/Me_4Si$): δ 185.2, 137.5, 128.0, 122.2, 105.5, 12.7, 11.7. MS (ESI^+): (*m/z*)[MH^+]124.08, 100%.

1,2,5-Trimethyl-1*H*-pyrrole-3-carbaldehyde (7l). [7, 8]

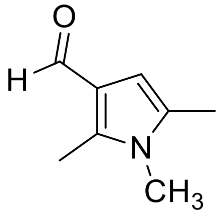

To an ice-cooled solution of 1,2,5-trimethylpyrrole (5 g, 0.046 mol) in dichloroethane (100 mL), was added DMF (4.18 g, 0.057 mol) at 0 ºC, and followed by dropwise addition of phosphorus oxychloride (8.43 g, 0.055 mol) at 0 ºC. The reaction mixture was allowed to stir at 0 ºC for 15 min and then heated at 80–85 ºC for 30 min. The mixture was cooled to room temperature and then poured into ice water, and made alkaline (pH 10–11) with aqueous solution of NaOH (20% w/v). The aqueous phase was extracted with (3 x 50 mL) of dichloromethane, and the combined organic phase was washed with water (2 x 100 mL), dried ($MgSO_4$) and concentrated *in vacuo* to yield a light brown solid (5.65 g, 90%), mp 94–95 ºC (lit. 96.5–97 ºC). ^{1}H NMR (500 MHz, $CDCl_3/Me_4Si$): δ 9.71 (s, 1H), 6.20 (s, 1H), 3.35 (s, 3H), 2.41 (s, 3H), 2.14 (s, 3H). ^{13}C NMR (125 MHz; $CDCl_3/Me_4Si$): δ 184.9, 138.1, 130.1, 121.3, 105.7, 30.1, 12.4, 10.7. MS (ESI^+): (*m/z*)[MH^+]138.09, 100%.

1-Benzyl-1H-pyrrole-3-carbaldehyde (7m)

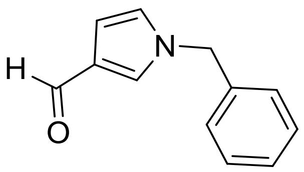

A mixture of 2,5-dimethoxytetrahydrofuran-3-aldehyde **8** (1.50 g, 0.0093 mol) and benzyl amine **5m** (0.832 g, 0.0078 mol) in glacial acetic acid (5 mL) was heated at 90 ºC for 12 h. The reaction mixture was cooled and poured into ice water and extracted with ethylacetate (2 x 100 mL). The organic layer was washed with water (3 x 50 mL) until neutral, dried ($MgSO_4$) and concentrated *in vacuo* to yield a dark brown liquid (1.45 g, 100 %). ^{1}H NMR (500 MHz, $CDCl_3/Me_4Si$): δ 9.76 (s, 1H), 7.37 (m, 4H), 7.19 (m, 2H), 6.73 (m, 1H), 6.69 (m, 1H), 5.12 (s, 2H). ^{13}C NMR (125 MHz, $CDCl_3/Me_4Si$): δ 185.4, 136.1, 129.2, 129.1 (2C), 128.4, 127.4 (2C), 126.9, 123.8, 108.7, 54.0. MS (ESI$^+$): (*m/z*)[MH$^+$]186.10, 100%.

C. General Condensation Procedure for cyclohexyl-2-(phenylimino)-4-thiazolidineionederivatives; 3-methyl-2-(phenylimino)thiazolidine-4-one derivatives; 3-methyl-2-(methylimino)thiazolidine-4-one derivatives thiazolidine-2,4-dione derivatives; 3-methyl thiazolidine-2,4-dione derivatives and imidazolidine-2,4-dionederivatives (20–54).

To a solution of cyclohexyl-2-(phenylimino)-4-thiazolidineione, 3-methyl-2-(phenylimino) thiazolidine-4-one, thiazolidine-2,4-dione, 3-methyl thiazolidine-2,4-dione or imidazolidine-2,4-dione (1.0 equiv) in absolute ethanol (15 mL) was added the requisite 2, 5-dimethyl-1-aryl-3-formylpyrrole (1.1 equiv), piperidine (1.2 equiv) and 3Å molecular sieves. The mixture was heated to reflux at 90 ºC for 8 h. After this time, the solution was filtered and concentrated *in vacuo*. The residue was purified by column chromatography on a silica gel column (ethylacetate/hexane) and recrystallized from methanol to give a yellowish solid product.

(2*Z*,5*Z*)-3-Cyclohexyl-5-((2,5-dimethyl-1-phenyl)-1*H*-pyrrol-3-yl)methylene-2-(phenylimino)thiazolidin-4-one (22).

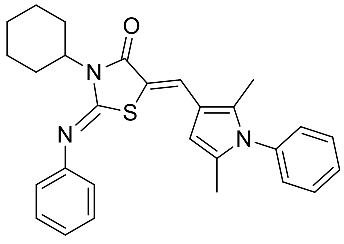

Yellowish solid, (0.330 g, 40%), mp. 198–199 ºC. ^{1}H NMR (500 MHz, $CDCl_3/Me_4Si$): δ 7.61 (s, 1H, CH=C), 7.39 (m, 3H), 7.32 (t, 2H, *J*= 7.83 Hz), 7.09 (m, 3H), 6.95 (d, 2H, *J*= 7.40 Hz), 6.02 (s, 1H), 4.53 (tt, 1H, *J*= 3.73, 3.98 Hz), 2.45 (dq, 2H, *J*= 5.25, 5.82 Hz), 2.05 (s, 3H), 1.89 (s, 3H),

1.80 (d, 2H, *J*= 13.05 Hz), 1.71 (d, 2H, *J*= 10.85 Hz), 1.61 (d, 1H, *J*= 12.75 Hz), 1.33 (m, 2H), 1.20 (m, 1H). ^{13}C NMR (125 MHz, $CDCl_3/Me_4Si$): δ 167.8 (C=O), 151.6 (C=N), 149.2(C=C) (2C), 137.7 (2C), 134.5 (2C), 131.2, 129.4 (2C), 129.3, 128.6, 127.9 (2C), 124.4, 124.2, 121.4, 120.9, 115.7, 114.2, 105.4, 55.6, 28.5, 26.2, 25.3, 12.7, 11.1. Anal. Calc for $C_{28}H_{29}N_3OS$ (455.61): C, 73.01; H, 6.42; N, 9.22; found: C, 71.84; H, 6.37; N, 8.99. HRMS (*m/z*): [MH$^+$] calcd for $C_{28}H_{30}N_3OS$, 456.2110; found 456.2076.

(2*Z*,5*Z*)-3-Cyclohexyl-5-((2,5-dimethyl-1-(4-(trifluoromethyl)phenyl)-1*H*-pyrrol-3-yl)methylene-2-(phenylimino)thiazolidin-4-one (23).

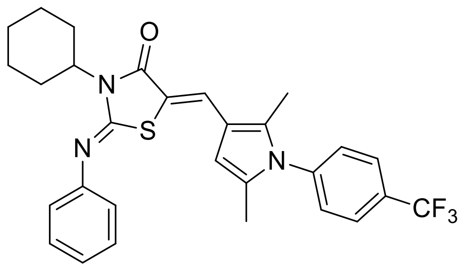

Yellowish solid, (0.190 g, 20%), mp. 218–220 ºC. ^{1}H NMR(500 MHz, $CDCl_3/Me_4Si$): δ 7.79 (d, 2H, *J*= 8.35 Hz), 7.68 (s, 1H, CH=C), 7.42 (dt, 2H, *J*= 3.16 Hz), 7.34 (d, 2H, *J*= 8.20 Hz), 7.20 (m, 1H), 7.05(m, 2H), 6.15 (s, 1H), 4.63 (tt, 1H, *J*= 3.80, 4.03 Hz), 2.54 (dq, 2H, *J* = 5.30, 5.85 Hz), 2.16 (s, 3H), 2.01 (s,3H), 1.91 (d, 2H, *J*= 13.41 Hz), 1.81 (d, 2H, *J*= 10.45 Hz), 1.71 (d, 1H, *J*= 12.65 Hz), 1.44 (m, 2H), 1.31 (m, 1H). ^{13}C NMR (125 MHz, $CDCl_3/Me_4Si$): δ 167.6 (C=O), 149.0 (C=C),, 133.9, 130.9 (2C), 130.7, 129.3 (2C), 128.5 (2C), 126.7 (2C), 124.8, 124.3, 123.8 (2C), 121.4 (2C), 116.3, 115.1, 106.1, 55.7, 28.5 (2C), 26.2 (2C), 25.3, 12.8, 11.1. Anal. Calc for $C_{29}H_{28}F_3N_3OS$ (523.61): C, 66.52; H, 5.39; N, 8.02; found: C, 66.17; H, 5.49; N, 7.90. HRMS (*m/z*): [MH$^+$] calcd for $C_{29}H_{29}F_3N_3OS$, 524.1983; found 524.1955.

(2*Z*,5*Z*)-3-cyclohexyl-5-((2,5-dimethyl-1-(3-(trifluoromethyl)phenyl)-1H-pyrrol-3-yl)methylene)-2-(phenylimino)thiazolidin-4-one (24).

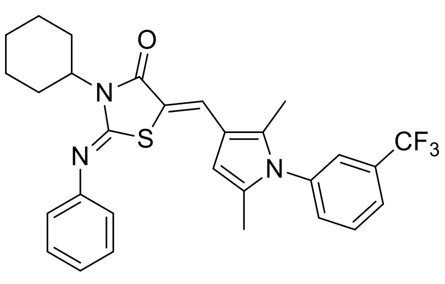

Yellowish solid, (0.190 g, 20%), mp. 124–125 ºC. ^{1}H NMR (500 MHz, $CDCl_3/Me_4Si$): δ 7.65 (d, 1H, *J*= 7.85 Hz), 7.58 (m, 2H), 7.39 (s, 1H, CH=C), 7.32 (m, 3H), 7.10 (t, 1H, *J*= 7.43 Hz), 6.95 (d, 2H, *J*=7.60 Hz), 6.05 (s, 1H), 4.53 (tt, 1H, *J*= 3.72, 3.98 Hz), 2.45 (dq, 2H, *J*= 5.23, 5.82 Hz), 2.06 (s, 3H), 1.90 (s, 3H), 1.80 (d, 2H, *J*= 13.20 Hz), 1.71 (d, 2H, *J*= 10.90 Hz), 1.61 (d, 1H, *J*= 12.60 Hz), 1.34 (m, 2H), 1.20 (m, 1H). ^{13}C NMR (125 MHz, $CDCl_3/Me_4Si$): δ 167.7(C=O), 151.4 (C=N),, 149.1(C=O) (2C), 138.4, 134.0 (2C), 132.2, 131.5, 130.9, 130.2, 129.3, 125.5, 125.0, 124.3 (2C), 123.9, 122.3, 121.4, 116.3, 115.1, 106.1, 55.7, 28.5, 26.2, 25.3, 22.7, 12.8, 11.1. Anal. Calc for $C_{29}H_{28}F_3N_3OS$ (523.61): C, 66.52; H, 5.39; N, 8.02; found: C, 63.58; H, 5.30; N, 7.43. HRMS (*m*/*z*): [MH^+] calcd for $C_{29}H_{29}F_3N_3OS$, 524.1983; found 524.1979.

(2*Z*,5*Z*)-3-Cyclohexyl-5-((2,5-dimethyl-1-(2-(trifluoromethyl)phenyl)-1*H*-pyrrol-3-yl)methylene-2-(phenylimino)thiazolidin-4-one (25).

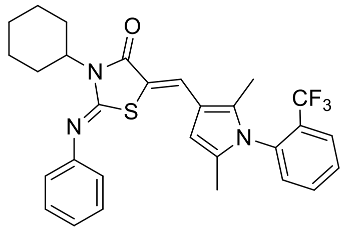

Yellowish solid, (0.210 g, 22%), mp. 125–127 ºC. ^{1}H NMR (500 MHz, $CDCl_3/Me_4Si$): δ 7.86 (dd, 1H, *J*= 1.22, 3.05 Hz), 7.72 (m, 1H), 7.69 (s, 1H, CH=C), 7.65 (t, 1H, *J*= 7.68 Hz), 7.42 (m, 2H), 7.26 (d, 1H, *J*= 7.70 Hz), 7.20 (m, 1H), 7.05 (m, 2H), 6.13 (s, 1H), 4.63 (tt, 1H, *J*= 3.82, 4.02 Hz), 2.55 (dq, 2H, *J*= 5.30, 5.87 Hz), 2.06 (s, 3H), 1.92 (d, 2H), 1.89 (s, 3H), 1.81 (d, 2H, *J*= 11.96 Hz), 1.70 (d, 1H, *J*= 12.00 Hz), 1.44 (m, 2H), 1.31 (m, 1H). ^{13}C NMR (125 MHz, $CDCl_3/Me_4Si$): δ 167.7(C=O), 151.6 (C=N), 149.2(C=C), 135.9, 135.6, 133.2, 132.0, 131.2, 130.9, 129.7, 129.3 (2C), 129.2, 128.9, 128.8, 127.6, 124.3, 121.6, 121.4, 116.0, 114.5, 105.5, 55.6, 28.4 (2C), 26.2, 25.3, 12.2, 10.8.Anal. Calc for $C_{29}H_{28}F_3N_3OS$ (523.61): C, 66.52; H, 5.39; N, 8.02; found: C, 65.73; H, 5.36; N, 7.77. HRMS (*m*/*z*): [MH^+] calcd for $C_{29}H_{29}F_3N_3OS$, 524.1983; found 524.1960.

(2*Z*,5*Z*)-3-Cyclohexyl-5-((2,5-dimethyl-1-(4-morpholinophenyl)-1*H*-pyrrol-3-yl)methylene)-2-(phenylimino)thiazolidin-4-one (26).

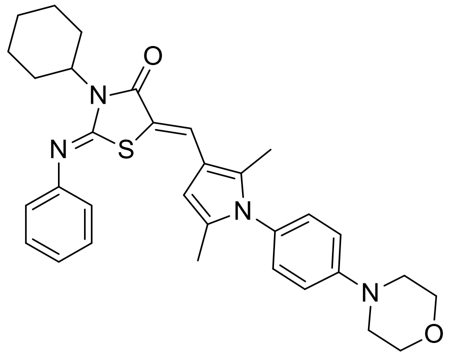

Yellowish solid, (0.065g, 12%), mp. 155–156 ºC. ^{1}H NMR (500 MHz, $CDCl_3/Me_4Si$): δ 7.78 (d, 1H, *J*= 3.75 Hz), 7.48 (m, 2H), 7.36 (d, 2H, *J*= 3.80 Hz), 7.26 (m, 1H), 7.13 (m, 3H), 7.04 (m, 1H), 6.16 (s, 1H), 4.69 (tt, 1H, *J*= 3.75, 6.16 Hz), 3.98 (m, 4H), 3.32 (m, 4H), 2.61(q, 2H, *J*= 12.44 Hz), 2.21 (d, 3H, *J*= 3.65 Hz), 2.05 (d, 3H, *J*= 3.00 Hz), 1.97 (d, 2H, *J* = 11.25 Hz), 1.87 (d, 2H, *J*= 11.65 Hz), 1.77 (d, 1H, *J*= 12.30 Hz), 1.51(m, 2H), 1.37 (m, 1H). ^{13}C NMR (125 MHz, $CDCl_3/Me_4Si$): δ 168.1(C=O), 150.7(C=N), 142.8(C=C),, 140.1, 131.7, 129.4 (2C), 128.7 (2C), 128.1, 124.7, 124.3, 121.5 (2C), 113.8, 115.6 (2C), 105.1 (2C), 100.2, 66.9 (2C), 55.6, 48.8 (2C), 28.5 (2C), 26.3, 25.4 (2C), 12.8, 11.1. Anal. Calc for $C_{32}H_{36}N_4O_2S$ (540.72): C, 71.08; H, 6.71; N, 10.36; found: C, 69.68; H, 6.62; N, 10.04. HRMS (*m/z*): [MH^+] calcd for $C_{32}H_{37}N_4O_2S$, 541.2637; found 541.2608.

(2*Z*,5*Z*)-3-Cyclohexyl-5-((2,5-dimethyl-1-(2-morpholinoethyl)-1*H*-pyrrol-3-yl)methylene)-2-(phenylimino)thiazolidin-4-one (27).

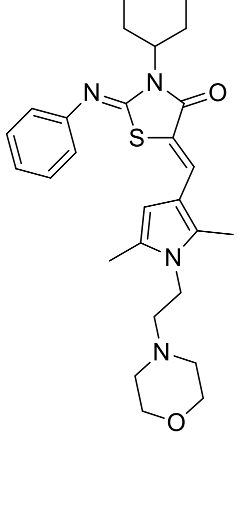

Yellowish solid, (0.132 g, 37%), mp. 168–169 ºC. ^{1}H NMR (500 MHz, $CDCl_3/Me_4Si$): δ 7.65 (s, 1H, CH=C), 7.41 (dt, 2H, *J*= 1.80, 3.15 Hz), 7.19 (tt, 1H, *J*= 1.12, 2.85 Hz), 7.03 (dd, 2H, *J*= 1.15, 2.38 Hz), 5.99 (s, 1H), 4.61 (tt, 1H, *J*= 3.80, 4.01 Hz), 3.91 (t, 2H, *J*= 7.28 Hz), 3.71 (t, 4H, *J*= 4.58 Hz), 2.52 (m, 4H), 2.47 (t, 4H, *J*= 4.43 Hz), 2.37 (s, 3H), 2.22 (s, 3H), 1.89 (d, 2H, *J*= 13.30 Hz), 1.79 (d, 2H, *J*= 10.55 Hz), 1.70 (d, 1H, *J*= 12.65 Hz), 1.43 (m, 2H), 1.31 (m, 1H). ^{13}C NMR (125 MHz, $CDCl_3/Me_4Si$): δ 167.8 (C=O), 151.7 (C=N), 149.2 (C=C), 133.4, 129.9, 129.3 (2C), 124.3, 124.2, 121.4 (2C), 115.4, 113.5, 105.5, 66.9 (2C), 58.5, 55.5, 54.1 (2C), 41.9, 28.4, 26.2 (2C), 25.3 (2C), 12.3, 10.4. Anal. Calc for $C_{28}H_{36}N_4O_2S$ (492.68): C, 68.26; H, 7.36; N, 11.37; found: C, 67.40; H, 7.35; N, 11.13. HRMS (*m*/*z*): [MH^+] calcd for C28H37N4O2S, 493.2637; found 493.2611.

(2*Z*,5*Z*)-3-Cyclohexyl-2-(phenylimino)-5-((1-(2-trifluoromethyl)phenyl)-1*H*-pyrrol-3-yl)methylene)thiazolidin-4-one (28).

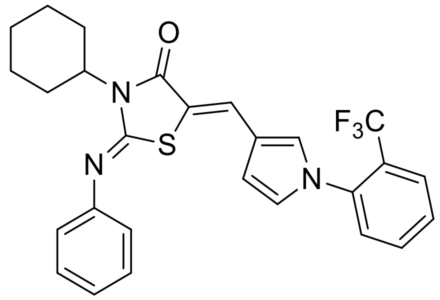

Yellowish solid, (0.095 g, 35%), mp. 145–147 ºC. ^{1}H NMR (500 MHz, $CDCl_3/Me_4Si$): δ 7.71 (d, 1H, *J*= 7.10 Hz), 7.57 (m, 2H, CH=C, Ar-H), 7.48 (t, 1H, *J*= 7.70 Hz), 7.30 (t, 3H, *J*= 3.95 Hz), 7.08 (t, 1H, *J*= 4.95 Hz), 6.98 (s, 1H), 6.92 (dd, 2H, *J*= 1.10, 3.10 Hz), 6.75 (t, 1H, *J*= 2.35 Hz), 6.38 (q, 1H, *J*= 1.55), 4.52 (tt, 1H, *J*= 3.80, 4.01 Hz), 2.53 (dq, 2H, *J*= 5.30, 5.87 Hz), 1.90 (d, 2H, *J*= 13.15 Hz), 1.80 (d, 2H, *J*= 10.80 Hz), 1.70 (d, 1H, *J*= 12.55 Hz), 1.44 (m, 2H), 1.31 (m, 1H). ^{13}C NMR (125 MHz, $CDCl_3/Me_4Si$): δ 167.2 (C=O), 150.9 (C=N), 148.9 (C=C), 138.3, 132.9, 129.4, 129.3 (2C), 128.8, 127.3, 126.9, 126.5, 125.4, 124.4 (2C), 124.0, 121.3 (2C), 120.6, 117.3, 110.3, 55.8, 28.4 (2C), 26.2 (2C), 25.2. HRMS (*m*/*z*): [MH^+] calcd for $C_{27}H_{25}F_3N_3OS$, 496.1670; found 496.1644.

(2*Z*,5*Z*)-5-((1-Benzyl-1H-pyrrol-3-yl)methylene)-3-cyclohexyl-2-(phenylimino)thiazolidin-4-one (29).

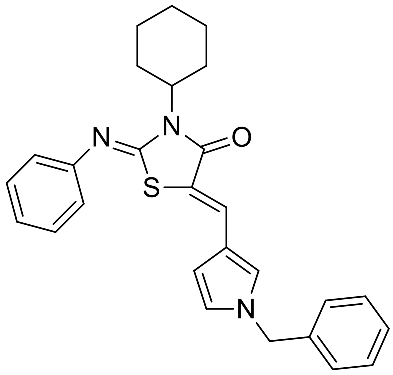

Yellowish solid, (0.143 g, 44%), mp. 68–70 ºC. ^{1}H NMR (500 MHz, $CDCl_3/Me_4Si$): δ 7.63 (s, 1H, CH=C), 7.40 (m, 2H), 7.34 (m, 3H), 7.19 (t, 1H, *J*= 7.43 Hz), 7.11 (dd, 2H, *J*= 1.50, 4.05 Hz), 7.02 (dd, 2H, *J*= 1.10, 3.03 Hz), 6.96 (t, 1H, *J*= 1.80 Hz), 6.68 (t, 1H, *J*= 2.48 Hz), 6.35 (m, 1H), 5.07 (s, 2H), 4.61 (tt, 1H, *J*= 3.80, 4.01 Hz), 2.53 (dq, 2H, *J*= 5.30, 5.87 Hz), 1.90 (d, 2H, *J*= 13.20 Hz), 1.79 (d, 2H, *J*= 10.80 Hz), 1.70 (d, 1H, *J*= 12.70 Hz), 1.43 (m, 2H), 1.31 (m, 1H). ^{13}C NMR (125 MHz, $CDCl_3/Me_4Si$): δ 167.4 (C=O), 151.1 (C=N), 149.0 (C=C),, 136.8, 129.3 (2C), 128.9 (2C), 128.1, 127.1 (2C), 125.12, 125.07, 124.4, 123.3, 121.3 (2C), 119.8, 115.9, 110.1, 55.7, 53.7, 28.4 (2C), 26.2 (2C), 25.2. Anal. Calc for $C_{27}H_{27}N_3OS$ (441.59): C, 73.44; H, 6.16; N, 9.51; found: C, 72.08; H, 6.03; N, 9.43. HRMS (*m*/*z*): [MH^+] calcd for $C_{27}H_{28}N_3OS$, 442.1953; found 442.1938.

(2*Z*,5*Z*)-3-Cyclohexyl-5-((2,5-dimethyl-1*H*-pyrrol-3-yl)methylene)-2-(phenylimino)thiazolidin-4-one (30).

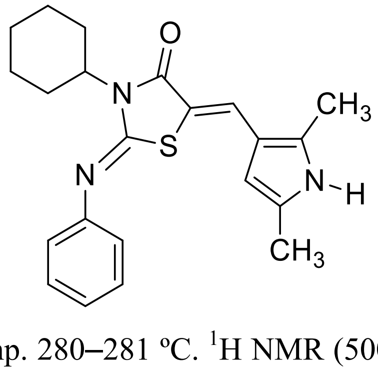

Yellowish solid, (0.173 g, 45%), mp. 280–281 ºC. ^{1}H NMR (500 MHz, $CDCl_3/Me_4Si$): δ 7.76 (brs, 1H, CH=C), 7.53 (s, 1H), 7.31 (dt, 2H, *J*= 1.80, 3.15 Hz), 7.09 (tt, 1H, *J*= 1.10, 2.83 Hz), 6.94 (dd, 2H, *J*= 1.15, 2.36 Hz), 5.86 (d, 1H, *J*= 1.40 Hz), 4.52 (tt, 1H, *J*= 3.82, 3.97 Hz), 2.44 (dq, 2H, *J*= 5.87 Hz), 2.27 (s, 3H), 2.11 (s, 3H), 1.80 (d, 2H, *J*= 13.31 Hz), 1.70 (d, 2H, *J*=6.45 Hz), 1.61 (d, 1H, *J*= 12.80 Hz), 1.33 (m, 2H), 1.20 (m, 1H). ^{13}C NMR (125 MHz; $CDCl_3/Me_4Si$): δ 167.8 (C=O),

149.2 (C=C), 132.2, 129.3 (2C), 124.2 (2C), 121.4 (2C), 116.1, 114.0, 113.1, 110.4, 105.3, 55.6, 28.4 (2C), 26.2, 25.3 (2C), 12.8, 11.4. Anal. Calc for $C_{22}H_{25}N_3OS$ (379.52): C, 69.63; H, 6.64; N, 11.07; found: C, 69.60; H, 6.67; N, 11.00. HRMS (*m/z*): [MH^+] calcd for $C_{22}H_{26}N_3OS$, 380.1797; found 380.1772.

(2*Z*,5*Z*)-3-Cyclohexyl-2-(phenylimino)-5-((1,2,5-trimethyl-1*H*-pyrrol-3-yl)methylene)thiazolidin-4-one (31).

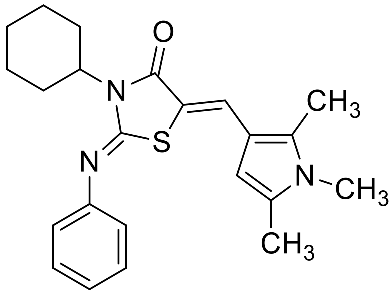

Yellowish solid, (0.067 g, 17%), mp. 184–185 ºC. ^{1}H NMR (500 MHz, $CDCl_3/Me_4Si$): δ 7.67 (s, 1H, CH=C), 7.41 (dt, 2H, *J*= 1.80, 3.94 Hz), 7.19 (t, 1H, *J*= 7.43 Hz), 7.03 (dd, 2H, *J*= 1.20, 3.14 Hz), 6.00 (s, 1H), 4.61 (tt, 1H, *J*= 3.85, 4.07 Hz), 3.42 (s, 3H, -NCH_3), 2.53 (dq, 2H, *J*= 5.43, 6.39 Hz), 2.33 (s, 3H), 2.19 (s, 3H), 1.89 (d, 2H, *J*= 13.35 Hz), 1.79 (d, 2H, *J*= 10.70 Hz), 1.70 (d, 1H, *J*= 13.60 Hz), 1.43 (m, 2H), 1.30 (m, 1H). ^{13}C NMR (125 MHz, $CDCl_3/Me_4Si$): δ167.9 (C=O), 149.2 (C=C), 133.9, 130.4 (2C), 129.3 (2C), 124.5, 124.2, 121.4 (2C), 114.9 (2C), 113.2 (2C), 104.9, 55.5, 30.6, 28.4, 26.2, 25.3, 12.3, 10.5. Anal. Calc for $C_{23}H_{27}N_3OS$ (393.54): C, 70.20; H, 6.91; N, 10.67; found: C, 63.64; H, 6.26; N, 9.47. HRMS (*m/z*): [MH^+] calcd for $C_{23}H_{28}N_3OS$, 394.1953; found 394.1952.

(2*Z*,5*Z*)-3-Cyclohexyl-2-(phenylimino)-5-((1-(2-trifluoromethyl)phenyl)-1*H*-pyrazol-4-yl)methylene)thiazolidin-4-one (**32**).

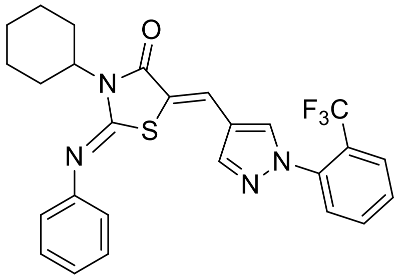

Yellowish solid, (0.090 g, 30%), mp. 60–61 ºC. ^{1}H NMR (500 MHz, $CDCl_3/Me_4Si$): δ 7.89 (s, 1H, CH=C), 7.82 (t, 2H, *J*= 3.43 Hz), 7.69 (t, 1H, *J*= 7.35 Hz), 7.65 (s, 1H), 7.61 (t, 1H, *J*= 7.65 Hz),

7.52 (d, 1H, *J*= 7.80 Hz), 7.40 (t, 2H, *J*= 7.85 Hz), 7.19 (t, 1H, *J*= 7.43 Hz), 7.02 (d, 2H, *J*= 4.15 Hz), 4.63 (tt, 1H, *J*= 3.75, 3.98 Hz), 2.53 (dq, 2H, *J*= 5.23, 5.83 Hz), 1.91 (d, 2H, *J*= 13.20 Hz), 1.81 (d, 2H, *J*= 11.00 Hz), 1.71 (d, 1H, *J*= 12.65 Hz), 1.44 (m, 2H), 1.30 (m, 1H). ^{13}C NMR (125 MHz, $CDCl_3/Me_4Si$): δ 166.6 (C=O), 149.7 (C=C), 148.6, 141.6, 137.8, 132.9, 132.3, 129.6, 129.4, 128.9, 127.4, 126.6, 126.2, 124.7, 123.9, 121.7, 121.1, 120.7, 120.1, 118.5, 55.9, 53.5, 28.4, 28.1, 26.2, 25.2Anal. Calc for $C_{26}H_{23}F_3N_4OS$ (496.55): C, 62.89; H, 4.67; N, 11.28; found: C, 58.79; H, 4.35; N, 10.20. HRMS (*m/z*): [MH$^+$] calcd for C26H24F3N4OS, 497.1623; found 497.1616.

(2*Z*,5*Z*)-5-((2,5-dimethyl-1-phenyl-1*H*-pyrrol-3-yl)methylene)-3-methyl-2-(phenylimino)thiazolidin-4-one(33).

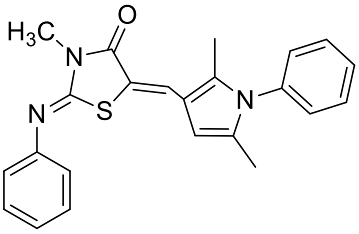

Yellowish solid, (0.188 g, 40 %), mp. 203–205 ºC. ^{1}H NMR (500 MHz, $CDCl_3/Me_4Si$): δ 7.69 (s, 1H, CH=C), 7.41 (m, 2H), 7.33 (m, 3H), 7.10 (m, 3H), 6.99 (m, 2H), 6.05 (s, 1H), 3.37 (s, 3H, -N-CH_3), 2.07 (s, 3H), 1.90 (s, 3H). ^{13}C NMR (125 MHz; $CDCl_3/Me_4Si$): δ 167.6 (C=O), 152.4 (C=N), 148.7 (C=C), 147.3, 137.6, 135.0, 132.9, 131.4, 129.5 (2C), 129.3, 128.7, 127.9, 125.1, 124.4, 121.5, 115.6, 113.9, 108.5, 105.3, 29.4, 12.7, 11.1. Anal. Calc for $C_{23}H_{21}N_3OS$ (387.5): C, 71.29; H, 5.46; N, 10.84; found: C, 70.51; H, 5.36; N, 10.74. HRMS (*m/z*): [MH$^+$] calcd for $C_{23}H_{21}N_3OS$, 388.1484; found 388.1463.

(2*Z*,5*Z*)-5-((2,5-Dimethyl-1-(2-(trifluoromethyl)phenyl-1*H*-pyrrol-3-yl)methylene)-3-methyl-2-(phenylimino)thiazolidin-4-one (34).

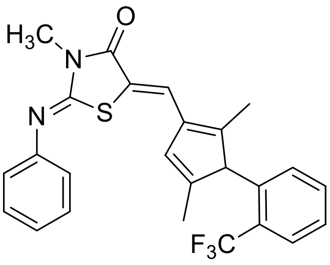

Yellowish solid, (0.200 g, 31%), mp. 207–208 ºC. ^{1}H NMR (500 MHz; $CDCl_3/Me_4Si$): δ 7.87 (dd, 1H, *J*= 1.20, 3.00 Hz), 7.76 (s, 1H, CH=C), 7.73(dt, 1H, *J*= 1.15, 3.31 Hz), 7.66 (t, 1H, *J*= 7.7 Hz), 7.43 (m, 2H), 7.26 (d, 1H, *J*= 7.7 Hz), 7.21 (tt, 1H, *J*= 1.15, 2.45 Hz), 7.08 (m, 2H, *J*= 1.60 Hz),

6.16 (s, 1H), 3.47 (s, 3H), 2.07 (s, 3H), 1.90 (s, 3H). ^{13}C NMR (125 MHz, $CDCl_3/Me_4Si$): δ 167.6 (C=O), 152.4 (C=N),, 148.8 (C=C), 143.2, 140.0, 135.9, 134.1, 133.2, 132.2, 131.2, 129.7, 129.4, 127.6, 125.0, 124.5, 121.5, 117.4, 115.9, 114.3, 105.4, 101.3, 29.5, 12.2, 10.8. Anal. Calc for $C_{24}H_{20}F_3N_3OS$ (455.5): C, 63.29; H, 4.43; N, 9.22; found: C, 62.79; H, 4.41; N, 9.15. HRMS (*m/z*): [MH^+] calcd for $C_{24}H_{21}F_3N_3OS$, 456.1354; found 456.1357.

(2*Z*,5*Z*)-5-((2,5-Dimethyl-1-(4-morpholinophenyl)-1*H*-pyrrol-3-yl)methylene)-3-methyl-2-(phenylimino)thiazolidin-4-one (35).

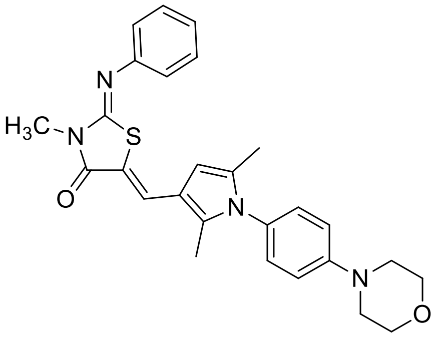

Yellowish solid, (0.262 g, 55%), mp. 288 ºC. ^{1}H NMR (500 MHz, $CDCl_3/Me_4Si$): δ 7.78 (s, 1H, CH=C), 7.42 (dt, 2H, *J*= 1.75, 3.16 Hz), 7.21 (tt, 1H, *J*= 1.05, 3.18 Hz), 7.08 (m, 4H), 6.98 (dd, 2H, *J*= 2.00, 4.48 Hz), 6.12 (s,1H), 3.91 (t, 4H, *J*= 4.83 Hz), 3.46 (s, 3H), 3.25 (t, 4H, *J* = 4.83 Hz), 2.16 (s, 3H), 1.99 (s, 3H). ^{13}C NMR (125 MHz, $CDCl_3/Me_4Si$): δ 167.7 (C=O), 152.5, 151.0 (C=N), 148.8 (C=C), 135.6, 131.8, 129.3 (2C), 129.2, 128.6, 125.3 (2C), 124.4 (2C), 121.5, 115.6, 115.3, 113.5 (2C), 105.0, 66.8 (2C), 48.7 (2C), 29.4, 12.7, 11.1. Anal. Calc for $C_{27}H_{28}N_4O_2S$ (472.60): C, 71.02; H, 6.18; N, 12.26; found: C, 66.44; H, 5.82; N, 11.33. HRMS (*m/z*): [MH^+] calcd for $C_{27}H_{29}N_4O_2S$, 473.2011; found 473.1984.

(2*Z*,5*Z*)-5-((2,5-Dimethyl-1-(2-morpholinoethyl)-1*H*-pyrrol-3-yl)methylene)-3-methyl-2-(phenylimino)thiazolidin-4-one (36).

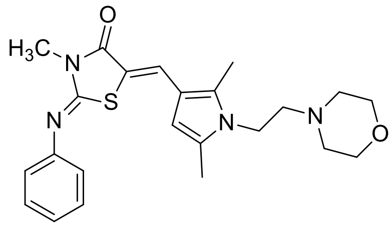

Yellowish solid, (0.175 g, 41%), mp. 199–200 ºC. ^{1}H NMR (500 MHz, $CDCl_3/Me_4Si$): δ 7.72 (s, 1H, CH=C), 7.41 (dt, 2H, *J*= 1.75, 3.15 Hz), 7.20 (tt, 1H, *J*= 1.07, 2.83 Hz), 7.06 (dd, 2H, *J*= 1.15, 3.15 Hz), 6.02 (s, 1H), 3.91 (t, 2H, *J*= 7.30 Hz), 3.71 (t, 4H, *J*= 4.58 Hz), 3.44 (s, 3H), 2.52 (t, 2H, *J*= 7.30 Hz), 2.48 (t, 4H, *J*= 4.35 Hz), 2.39 (s, 3H), 2.23 (s, 3H). ^{13}C NMR (125 MHz,

$CDCl_3/Me_4Si$): δ 167.7 (C=O), 152.4 (C=N), 148.8 (C=C), 133.9, 130.2, 129.3 (2C), 125.1, 124.4, 121.5 (2C), 115.3, 113.2, 105.5, 66.9 (2C), 58.5, 54.1 (2C), 41.9, 29.4, 12.3, 10.4. Anal. Calc for $C_{23}H_{28}N_4O_2S$ (424.56): C, 65.07; H, 6.65; N, 13.19; found: C, 64.91; H, 6.66; N, 13.05. HRMS (*m/z*): [MH^+] calcd for $C_{23}H_{29}N_4O_2S$, 425.2011; found 425.2024.

(2*Z*,5*Z*)-5-((1-Benzyl-1*H*-pyrrol-3-yl)methylene)-3-methyl-2-(phenylimino)thiazolidin-4-one (37).

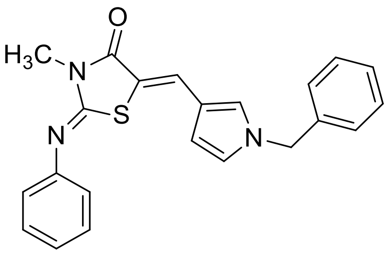

Yellowish solid, (0.199 g, 52%), mp. 155–156 ºC. ^1H NMR (500 MHz, $CDCl_3/Me_4Si$): δ 7.70 (s, 1H, CH=C), 7.41 (t, 2H, *J*= 7.85 Hz), 7.35 (m, 3H), 7.20 (t, 1H, *J*= 7.43 Hz), 7.13 (dd, 2H, *J*= 1.40, 4.03 Hz), 7.05 (dd, 2H, *J*= 1.10, 4.23 Hz), 6.99 (t, 1H, *J*= 1.83 Hz), 6.70 (t, 1H, *J*= 2.50 Hz), 6.36 (t, 1H, *J*=1.55 Hz), 5.08 (s, 2H), 3.45 (s, 3H). ^{13}C NMR (125 MHz, $CDCl_3/Me_4Si$): δ 167.31, 151.8, 148.6, 136.7, 129.4 (2C), 128.9 (2C), 128.2, 127.1 (2C), 125.8 (2C), 125.4, 124.6, 123.4, 121.4 (2C), 119.6, 115.6, 110.1, 29.5. Anal. Calc for $C_{22}H_{19}N_3OS$ (373.47): C, 70.75; H, 5.13; N, 11.25; found: C, 70.53; H, 5.04; N, 11.23. HRMS (*m/z*): [MH^+] calcd for $C_{22}H_{20}N_3OS$, 374.1327; found 374.1293.

(2*Z*,5*Z*)-3-Methyl-2-(phenylimino)-5-(1-(2-(trifluoromethyl)phenyl-1*H*-pyrazol-3-yl)methylene)thiazolidin-4-one (38).

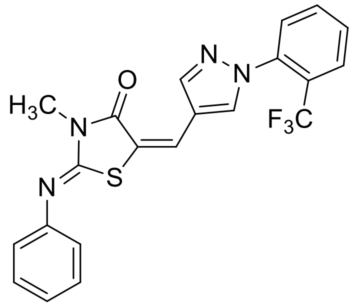

Yellowish solid, (0.166 g, 32%), mp. 135 ºC. ^1H NMR (500 MHz, $CDCl_3/Me_4Si$): δ 7.91 (s, 1H, CH=C), 7.84 (m, 1H), 7.71 (m, 2H), 7.63 (m, 2H), 7.54 (m, 1H), 7.41 (t, 2H, *J*= 7.88 Hz), 7.21 (m, 1H), 7.05 (dd, 2H, *J*= 1.20, 3.15 Hz), 3.49 (s, 3H, -N-CH_3). ^{13}C NMR (125 MHz, $CDCl_3/Me_4Si$): δ

166.6 (C=O), 150.4 (C=N), 148.1 (C=C), 137.7, 132.9, 132.5, 132.4, 129.6, 129.4, 128.9, 127.4, 126.3, 126.1, 124.9, 123.9, 121.7, 121.1, 120.9, 120.4, 118.3, 29.8. Anal. Calc for C21H15F3N4OS (428.43): C, 58.87; H, 3.53; N, 13.07; found: C, 58.88; H, 3.43; N, 13.03. HRMS (*m/z*): [MH$^+$] calcd for C21H16F3N4OS, 429.0997; found 429.0994.

(2*Z*,5*Z*)-5-((2,5-Dimethyl-1-(4-morpholinophenyl)-1*H*-pyrrol-3-yl)methylene)-3-methyl-2-(methylimino)thiazolidin-4-one (39).

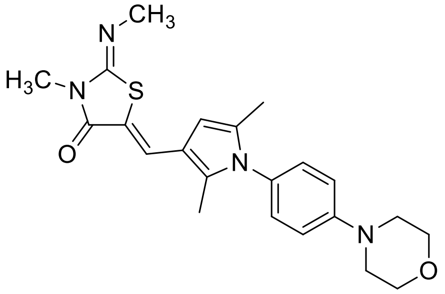

Yellowish solid, (0.120 g, 29%), mp. 333–334 ºC. ^{1}H NMR (500 MHz, $CDCl_3/Me_4Si$): *δ* 7.74 (s, 1H, CH=C), 7.11 (d, 2H, *J* = 8.85 Hz), 7.00 (d, 2H, *J* = 8.90 Hz), 6.31 (s, 1H), 3.92 (t, 4H, *J* = 4.80 Hz), 3.31 (s, 3H), 3.28 (s, 3H), 3.26 (t, 4H, J= 4.85 Hz), 2.18 (s, 3H), 2.07 (s, 3H). ^{13}C NMR (125 MHz, $CDCl_3/Me_4Si$): *δ* 167.6 (C=O), 152.3 (C=N), 151.0 (C=C), 135.3, 131.7, 129.3, 128.6 (2C), 124.3, 115.6 (2C), 115.4, 113.7, 104.9, 66.8 (2C), 48.8 (2C), 39.1, 29.1, 12.9, 11.1. Anal. Calc for $C_{22}H_{26}N_4O_2S$ (410.53): C, 64.37; H, 6.38; N, 13.64; found: C, 63.17; H, 6.26; N, 13.29. HRMS (*m/z*): [MH$^+$] calcd for $C_{22}H_{27}N_4O_2S$, 411.1855; found 411.1839.

(2*Z*,5*Z*)-5-((2,5-Dimethyl-1-(2-morpholinoethyl)-1*H*-pyrrol-3-yl)methylene-3-methyl-2-(methylimino)thiazolidin-4-one (40).

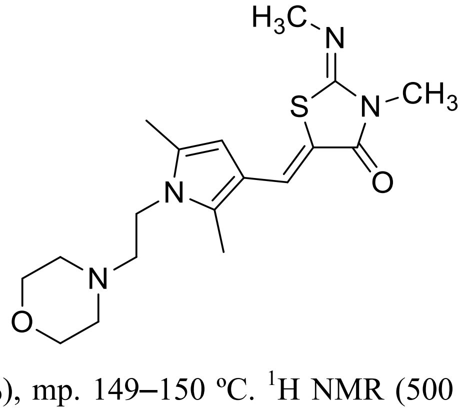

Yellowish solid, (0.300 g, 83%), mp. 149–150 ºC. ^{1}H NMR (500 MHz, $CDCl_3/Me_4Si$): *δ* 7.69 (s, 1H, CH=C), 6.22 (s, 1H), 3.95 (t, 2H, *J* = 7.33 Hz), 3.73 (t, 4H, *J* = 4.58 Hz), 3.29 (s, 3H), 3.26 (s, 3H), 2.56 (t, 2H, *J* = 7.35 Hz), 2.51 (t, 4H, *J* = 4.30 Hz), 2.41 (s, 3H), 2.32 (s, 3H). ^{13}C NMR (125

MHz, $CDCl_3/Me_4Si$): *δ* 167.6 (C=O), 152.6 (C=C), 133.6, 130.1, 124.1, 115.4, 113.5, 105.5, 66.9 (2C), 58.5, 54.1 (2C), 41.9, 39.1, 29.1, 12.5, 10.4. Anal. Calc for $C_{18}H_{26}N_4O_2S$ (362.49): C, 59.64; H, 7.23; N, 15.45; found: C, 59.37; H, 7.30; N, 15.51. HRMS (*m/z*): [MH^+] calcd for $C_{18}H_{27}N_4O_2S$, 363.1855; found 363.1851.

(2*Z*,5*Z*)-3-Methyl-2-(methylimino)-5-((1-(2-trifluoromethyl)phenyl)-1*H*-pyrrol-3-yl)methylene)thiazolidin-4-one (**41**).

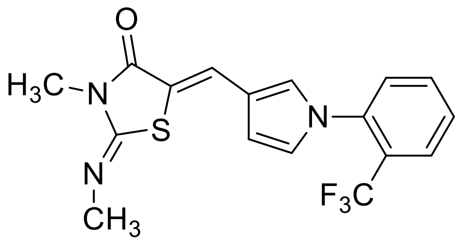

Yellowish solid, (0.120 g, 33%). mp. 54–55 ºC. ^1H NMR (500 MHz, $CDCl_3/Me_4Si$): *δ* 7.83 (d, 1H, *J*= 7.85 Hz), 7.71 (s, 1H, CH=C), 7.68 (dd, 1H, *J*= 0.96, 2.87 Hz), 7.60 (t, 1H, *J*= 7.70 Hz), 7.45 (d, 1H, *J*= 7.80 Hz), 7.18 (s, 1H), 6.93 (m, 1H), 6.61 (q, 1H, *J*= 1.55 Hz), 3.30 (s, 3H), 3.26 (s, 3H). ^{13}C NMR (125 MHz, $CDCl_3/Me_4Si$): *δ* 167.1 (C=O), 151.2 (C=C),, 138.3, 132.9, 129.4, 128.9, 127.3, 126.7, 126.6, 125.5, 124.3, 121.9, 120.6, 117.1, 110.2, 39.2, 29.2. Anal. Calc for $C_{17}H_{14}F_3N_3OS$ (365.37): C, 55.88; H, 3.86; N, 11.50; found: C, 54.95; H, 3.88; N, 11.39. HRMS (*m/z*): [MH^+] calcd for $C_{17}H_{15}F_3N_3OS$, 366.0888; found 366.0881.

(2*Z*,5*Z*)-5-((1-Benzyl-1*H*-pyrrol-3-yl)methylene-3-methyl-2-(methylimino)thiazolidin-4-one (42).

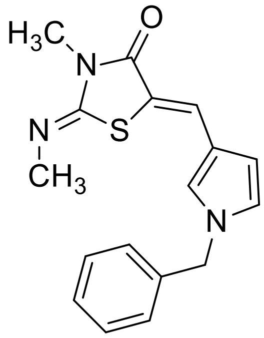

Yellowish solid, (0.128 g, 41%), mp. 128–130 ºC. ^1H NMR (500 MHz, $CDCl_3/Me_4Si$): *δ* 7.67 (s, 1H, CH=C), 7.37 (m, 3H), 7.17 (d, 2H, *J*= 6.95 Hz), 7.06 (t, 1H, *J*= 1.82 Hz), 6.77 (t, 1H, *J*= 2.50 Hz), 6.49 (t, 1H, *J*= 2.33 Hz), 5.13 (s, 2H), 3.28 (d, 6H, *J*= 17.35 Hz). ^{13}C NMR (125 MHz;

$CDCl_3/Me_4Si$): δ 167.4 (C=O), 151.8 (C=C),, 136.7, 129.0 (2C), 128.2, 127.2 (2C), 125.3, 124.9, 123.4, 119.8, 115.8, 109.9, 53.8, 39.2, 29.2. Anal. Calc for $C_{17}H_{17}N_3OS$ (311.40): C, 65.57; H, 5.50; N, 13.49; found: C, 65.37; H, 5.44; N, 13.55. HRMS (*m/z*): [MH^+] calcd for $C_{17}H_{18}N_3OS$, 312.1171; found 312.1161.

(2*Z*,5*Z*)-3-Methyl-2-(methylimino)-5-((1-(2-trifluoromethyl)-phenyl)-1*H*-pyrazol-4-yl)methylene)thiazolidin-4-one (**43**).

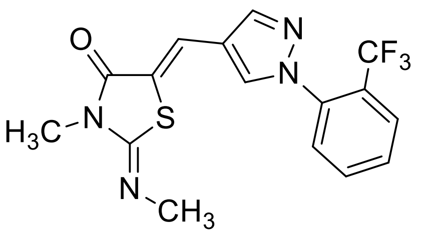

Yellowish solid, (0.129 g, 35%), mp. 170–172 ºC. ^{1}H NMR (500 MHz, $CDCl_3/Me_4Si$): δ 8.01 (s, 1H, CH=C), 7.96 (s, 1H), 7.87 (dd, 1H, J= 1.00, 3.95 Hz), 7.74 (dt, 1H, J= 1.05, 3.28 Hz), 7.69 (s, 1H), 7.65 (t, 1H, J= 7.7 Hz), 7.60 (d, 1H, J= 7.9 Hz), 3.31 (d, 6H, N-CH_3, J= 21.11 Hz). ^{13}C NMR (125 MHz, $CDCl_3/Me_4Si$): *δ* 166.5 (C=O), 149.8 (C=C), 141.6, 137.9, 133.0, 132.5, 129.6. 128.9, 127.4, 126.0, 124.0, 120.4, 119.9, 118.6, 39.3, 29.4. Anal. Calc for $C_{16}H_{13}F_3N_4OS$ (366.36): C, 52.46; H, 3.58; N, 15.29; found: C, 51.50; H, 3.58; N, 14.51. HRMS (*m/z*): [MH^+] calcd for $C_{16}H_{14}F_3N_4OS$, 367.0840; found 367.0851.

(*Z*)-5-((2,5-Dimethyl-1-(2-(trifluoromethyl)phenyl)-1*H*-pyrrol-3-yl)methylene)thiazolidine-2,4-dione (44).

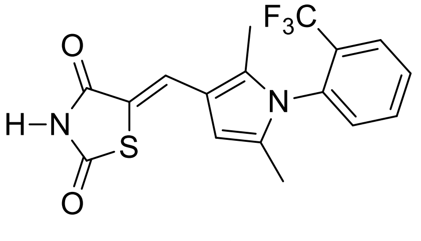

Yellowish solid, (0.117 g, 15%), mp. 234–235 ºC. ^{1}H NMR (500 MHz; $CDCl_3/Me_4Si$): δ 8.70 (brs, 1H, CH=C), 7.90 (d, 1H, J= 7.10 Hz), 7.87 (s, 1H), 7.76 (dt, 1H, J= 1.05, 3.26 Hz), 7.69 (t, 1H, J= 7.68 Hz), 7.30 (s, 1H), 6.23 (s, 1H), 2.10 (s, 3H), 1.97 (s, 3H). ^{13}C NMR (125 MHz; $CDCl_3/Me_4Si$): δ 168.1 (C=O), 167.1 (C=O), 137.9 (C=C),, 135.6, 133.4, 133.1, 131.0, 129.9, 128.6 (2C), 127.6, 123.7, 115.8, 114.5, 105.4, 12.3, 10.9. Anal. Calc for $C_{17}H_{13}F_3N_2O_2S$ (366.36): C, 55.73; H, 3.58; N, 7.64; found: C, 55.33; H, 3.59; N, 7.49. HRMS (*m/z*): [MH^+] calcd for $C_{17}H_{14}F_3N_2O_2S$, 367.0728, found 367.0734.

(*Z*)-5-((2,5-Dimethyl-1-phenyl)-1*H*-pyrrol-3-yl)methylene)-3-methylthiazolidine-2,4-dione (45).

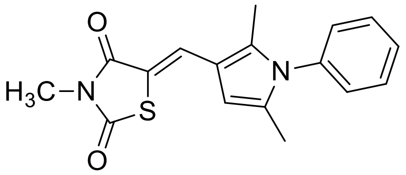

Yellowish solid, (0.078 g, 33%), mp. 195–196 ºC. ^{1}H NMR (500 MHz, $CDCl_3/Me_4Si$): δ 7.83 (s, 1H, CH=C), 7.43 (m, 3H), 7.12 (m, 2H), 6.14 (s, 1H), 3.15 (s, 3H), 2.10 (s, 3H), 1.96 (s, 3H). ^{13}C NMR (125 MHz, $CDCl_3/Me_4Si$): δ 168.9 (C=O), 167.1 (C=O),, 137.4 (C=C),, 136.4, 132.1 (2C), 129.6 (2C), 128.9, 128.0, 127.9, 115.6, 113.3, 105.3, 27.7, 12.9, 11.2. Anal. Calc for $C_{17}H_{16}N_2O_2S$ (312.39): C, 62.18; H, 4.91; N, 8.53; found: C, 64.33; H, 5.08; N, 8.71. HRMS (*m/z*): [MH^+] calcd for $C_{17}H_{17}N_2O_2S$, 313.1011; found 313.0992.

(*E*)-5-((2,5-Dimethyl-1-(2-(trifluoromethyl)phenyl)-1*H*-pyrrol-3-yl)methylene)thiazolidine-2,4-dione (46).

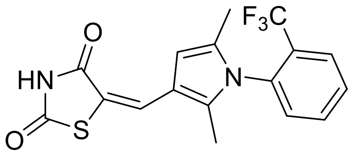

Yellowish solid, (0.099 g, 72%), mp. 205–210 ºC. ^{1}H-NMR (500 MHz, DMSO-d_6): δ 12.41 (s, 1H, -NH), 8.02-8.01 (d, 1H, *J* = 7.35 Hz), 7.94-7.91 (t, 1H, *J* = 7.00, 7.60 Hz), 7.84-7.81 (t, 1H, *J* = 7.75, 7.65 Hz), 7.65 (s, 1H, CH=C), 7.54-7.52 (t, 1H, *J* = 7.75Hz), 6.20 (s, 1H), 3.35 (s, 1H), 2.02 (s, 3H), 1.89 (s, 3H).^{13}C-NMR (125 MHz, DMSO-d_6): δ 168.2 (C=O), 153.2, 136.8 (2C), 134.4 (2C), 132.4, 131.6 (2C), 130.6 (2C), 125.8 (2C), 114.8, 104.7, 11.9, 10.4; HRMS (*m/z*): [MH^+] calcd for $C_{17}H_{14}F_3N_2O_2S$, 367.0711; found: 367.0715.

(*E*)-5-((2, 5-dimethyl-1-(2-(trifluoromethyl)phenyl)-1*H*-pyrrol-3-yl)methylene)-2-iminothiazolidin-4-one (47).

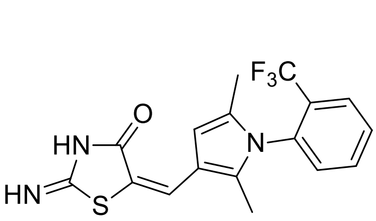

Yellowish solid, (0.098 g, 72%), mp. 285–290 ºC. ^{1}H-NMR (500 MHz, DMSO-d_6): δ 8.87 (s, 1H, -NH), 8.02-8.00 (d, 1H, *J* = 7.45 Hz), 7.93-7.90 (t, 1H, *J* = 7.7, 7.3 Hz), 7.83-7.80 (t, 1H, *J* = 7.75

Hz), 7.52-7.51 (t, 1H, J = 7.75 Hz, CH=C), 7.47-7.46 (d, 1H, J = 7.45 Hz), 6.14 (s, 1H), 3.35, (s, 1H), 1.98 (s, 3H), 1.91-1.89 (d, 3H, J = 9.8 Hz). ^{13}C-NMR (125 MHz, DMSO-d$_6$): δ 174.9 (C=O), 171.9 (C=N), 166.3, 134.8, 134.3, 131.7, 131.6, 130.6, 127.5, 122.9, 122.2, 117.5, 115.3, 104.6, 21.0, 11.9, 10.3. HRMS (m/z): [MH$^+$] calcd for $C_{17}H_{15}F_3N_3OS$, 366.0882; found: 366.0881.

(*E*)-5-((2,5-Dimethyl-1-(2-(trifluoromethyl)phenyl)-1*H*-pyrrol-3-yl)methylene)-2-thioxothiazolidin-4-one (48).

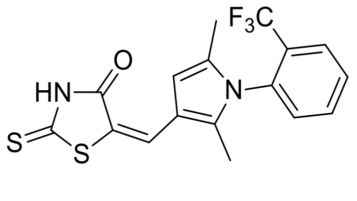

Yellowish solid, (0.130 g, 90%), mp 255–258 ºC. ^{1}H-NMR (500 MHz, DMSO-d$_6$): δ 13.51 (s, 1H, -NH), 8.03-8.02 (d, 1H,J = 0.9, 6.9 Hz), 7.94-7.91(t, 1H,J = 0.9, 6.9 Hz), 7.84-7.81 (t, 1H, J = 7.7 Hz), 7.56-7.54 (t, 1H,J = 7.75 Hz), 7.40 (s, 1H, CH=C), 6.22 (s, 1H), 2.04 – 2.03 (d, 3H, J = 6.95 Hz), 1.91-1.87 (t, 3H, J = 8.43, 13.8 Hz). ^{13}C-NMR (125 MHz, DMSO-d$_6$): δ 195.1 (C=S), 169.2 (C=O), 166.3, 138.4, 138.5, 134.5, 133.2, 131.5, 130.7, 127.6, 127.6, 126.1, 117.5, 115.5, 105.1, 11.9, 10.4. HRMS (m/z): [MH$^+$] calcd for $C_{17}H_{14}F_3N_2OS_2$, 383.0494; found: 383.0480.

(*Z*)-5-((2,5-Dimethyl-1-(2-(trifluoromethyl)phenyl)-1*H*-pyrrol-3-yl)methylene)imida zolidine-2,4-dione (49).

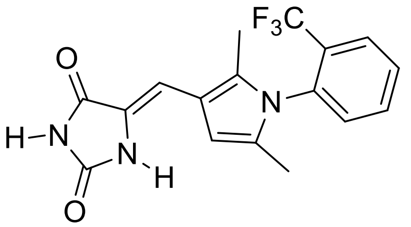

Yellowish solid, (0.077 g, 22%), mp. 265–267 ºC. ^{1}H NMR (500 MHz, CD$_3$OD): δ 9.67 (s, 1H, -NH), 7.84 (d, 1H, J= 7.85 Hz), 7.73 (t, 1H, J= 7.63 Hz), 7.65 (t, 1H, J= 7.63 Hz), 7.27 (d, 1H, J= 7.65 Hz), 6.55 (s, 1H, CH=C), 6.34 (s, 1H), 4.80 (s, 1H, -NH), 1.90 (s, 3H), 1.88 (s, 3H). ^{13}C NMR (125 MHz, CD$_3$OD): δ 168.1 (C=O), 157.4 (C=O), 137.4, 135.4, 134.9 (2C), 133.1, 132.9 (2C), 131.2, 128.6, 124.1, 115.3, 107.9, 106.3, 12.4, 10.6. Anal. Calc for $C_{17}H_{14}F_3N_3O_2$ (349.31): C, 58.45; H, 4.04; N, 12.02; found: C, 54.54; H, 3.75; N, 10.69. HRMS (m/z): [MH$^+$] calcd for $C_{17}H_{14}F_3N_3O_2$, 350.1116; found: 350.1092.

(*Z*)-5-((2,5-Dimethyl-1-phenyl)-1*H*-pyrrol-3-yl)methylene)imidazolidine-2,4-dione (50).

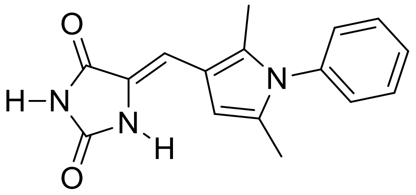

Yellowish solid, (0.127 g, 45%), mp. 267–270 ºC decompose. ^{1}H NMR (500 MHz; $CDCl_3/Me_4Si$): δ 9.81 (s, 1H, -NH), 7.42 (m, 3H), 7.12 (m, 2H), 6.70 (s, 1H, CH=C), 6.05 (s, 1H), 2.04 (s, 3H), 1.98 (s, 3H). ^{13}C NMR (125 MHz; $CDCl_3/Me_4Si$): δ 162.5 (C=O), 153.5 (C=O), 133.8, 131.6, 129.5, 127.9 (2C), 122.1, 117.7, 113.4, 108.1, 104.4, 60.4, 14.2, 12.8, 11.0. Anal. Calc for $C_{16}H_{15}N_3O_2$(281.31): C, 68.31; H, 5.37; N, 14.93; found: C, 65.05; H, 5.23; N, 13.09. HRMS (*m/z*): [MH^+] calcd for $C_{16}H_{16}N_3O_2$, 282.1243; found: 282.1230.

5-((2,5-Dimethyl-1-(2-(trifluoromethyl)phenyl)-1*H*-pyrrol-3-yl)methylene)pyrimidine-2,4,6(1*H*,3*H*,5*H*)-trione (51).

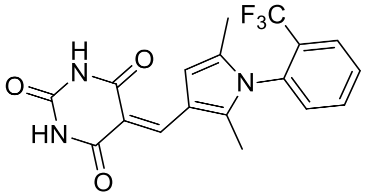

Yellowish solid, (0.108 g, 73%), mp. >300 ºC. ^{1}H-NMR (500 MHz, DMSO-d_6): δ 11.06 (d, 1H, -NH, J = 1.5 Hz), 10.93 (d, 1H, -NH, J = 1.5 Hz), 8.29 (s, 1H, CH=C), 8.06-8.04 (d, 1H,J = 7.7 Hz), 7.97-7.94 (d, 1H,J = 7.7 Hz), 7.87-7.84 (t, 1H, J = 7.7 Hz), 7.61-7.60 (d, 1H,J = 6.4 Hz), 7.51(d, 1H, J = 0.55Hz), 2.51 – 2.50 (m, 3H), 2.14 (s, 3H), 1.89 (d, 3H, J = 0.3 Hz). ^{13}C-NMR (125 MHz, DMSO-d_6): δ 164.7, 162.6, 145.9, 134.1, 134.1, 131.3, 130.9, 127.7, 127.7, 126.5, 123.9, 121.7, 119.5, 117.7, 110.7, 108.2, 11.9, 11.1; HRMS (*m/z*): [MH^+] calcd for $C_{18}H_{15}F_3N_3O_3$, 378.1069; found: 378.1064.

5-((2,5-Dimethyl-1-(2-(trifluoromethyl)phenyl)-1*H*-pyrrol-3-yl)methylene)-2-thioxodihydropyrimidine-4,6(1*H*,5*H*)-dione (52).

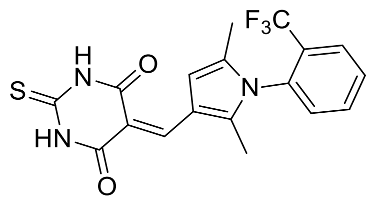

Yellowish solid, (0.107 g, 71%), mp. >300 ºC. ^{1}H-NMR (500 MHz, DMSO-d$_6$): δ 12.18 (brs, 1H, -NH), 12.09 (brs, 1H, -NH), 8.30 (s, 1H, CH=C), 8.07-8.05 (d, 1H, J = 7.8 Hz), 7.98-7.95 (d, 1H, J = 7.0 Hz), 7.88-7.85 (t, 1H, J = 7.7 Hz), 7.63-7.62 (d, 1H, J = 7.8 Hz), 7.75 (brs, 1H), 2.16 (s, 3H), 1.90 (s, 3H). ^{13}C-NMR (125 MHz, DMSO-d$_6$): δ 177.9, 169.2, 163.0, 166.3, 160.4, 147.8, 146.9, 134.6, 133.9, 132.8, 131.2, 131.0, 130.9, 118.4, 110.8, 108.5, 11.9, 11.3. HRMS (*m/z*): [MH^{+}] calcd for $C_{18}H_{15}F_3N_3O_2S$, 394.0832; found: 394.0831.

III. NMR Spectra

Compound 20_H-NMR

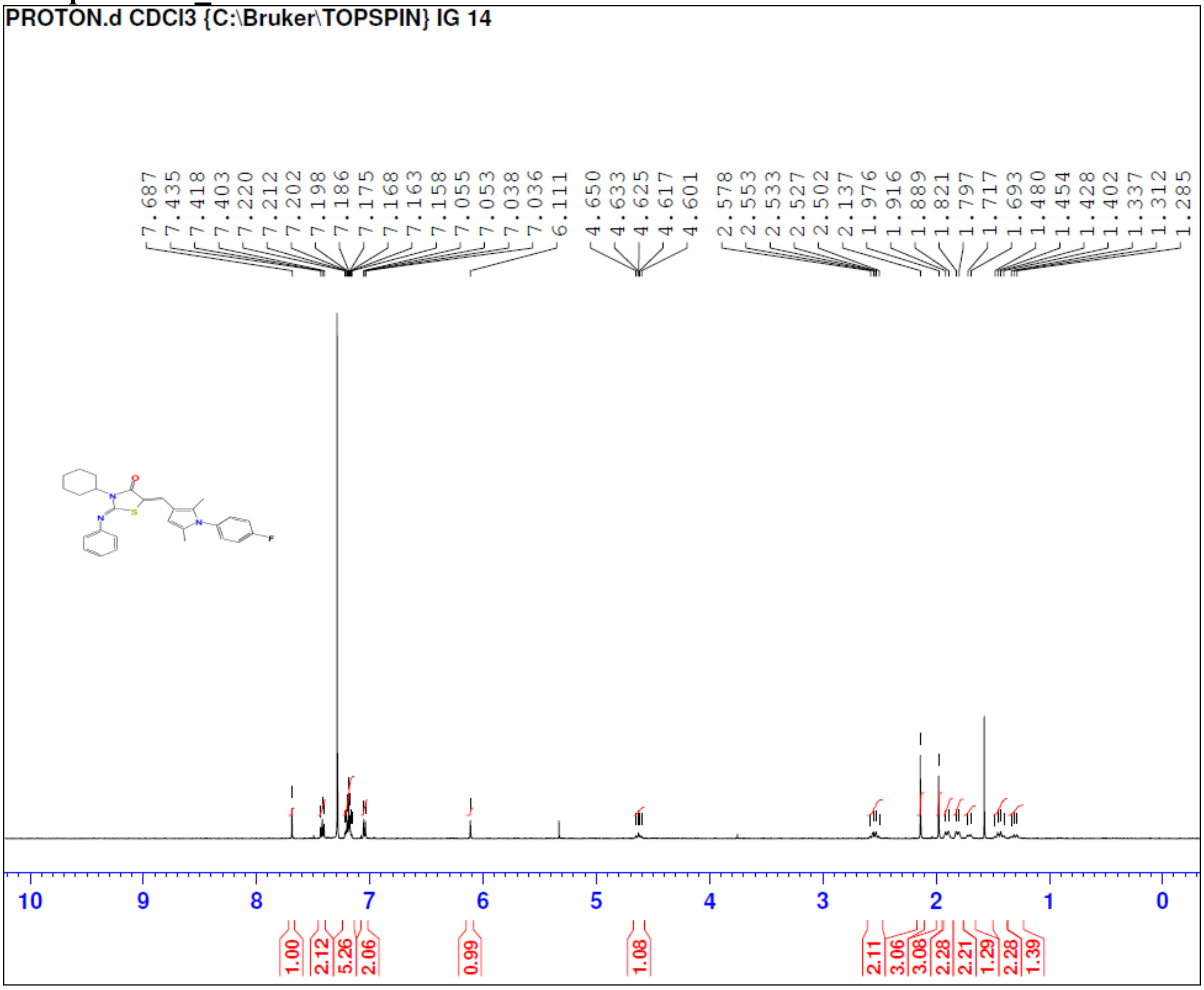

Compound 20_C-NMR

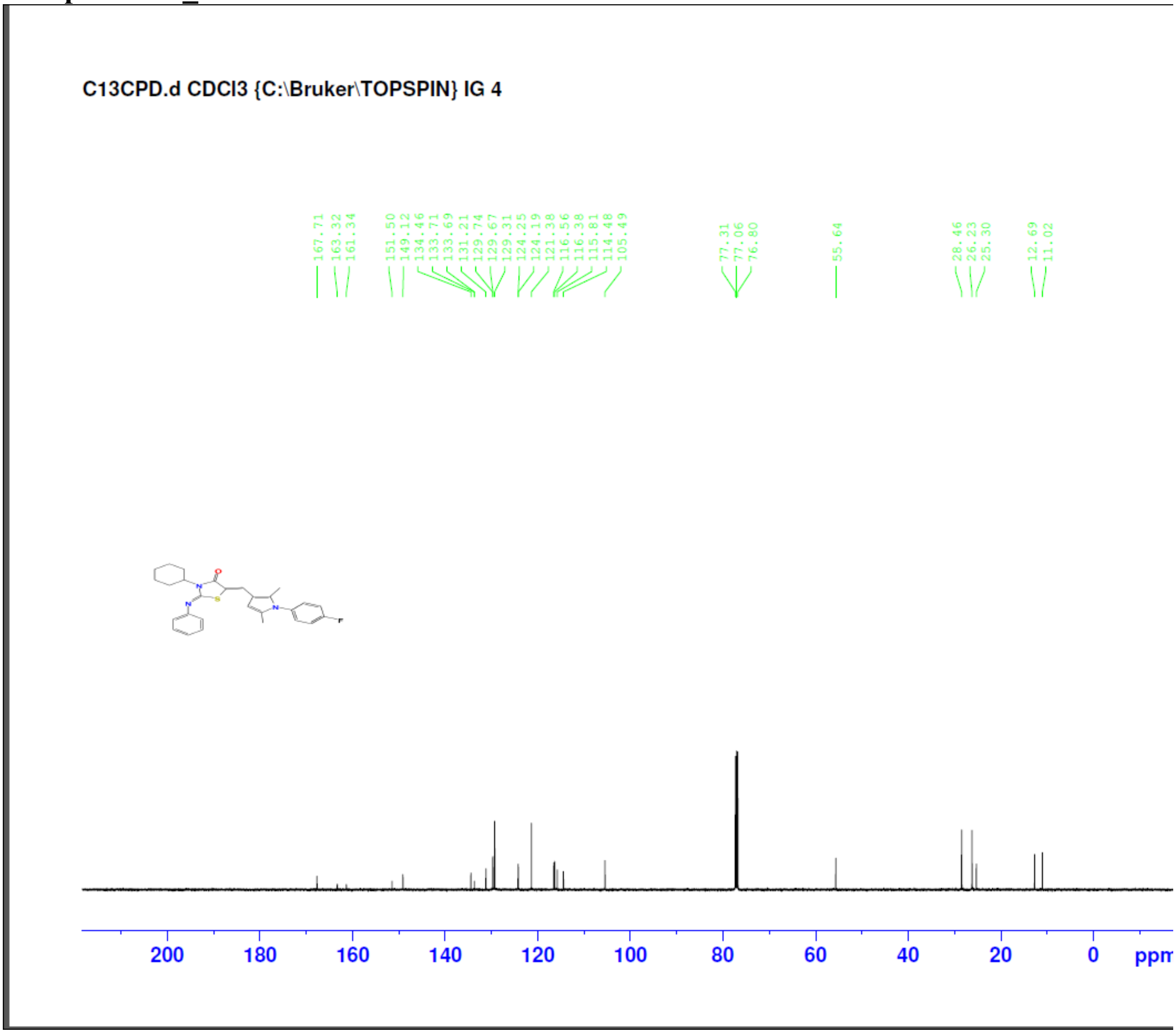

Compound 23_H-NMR

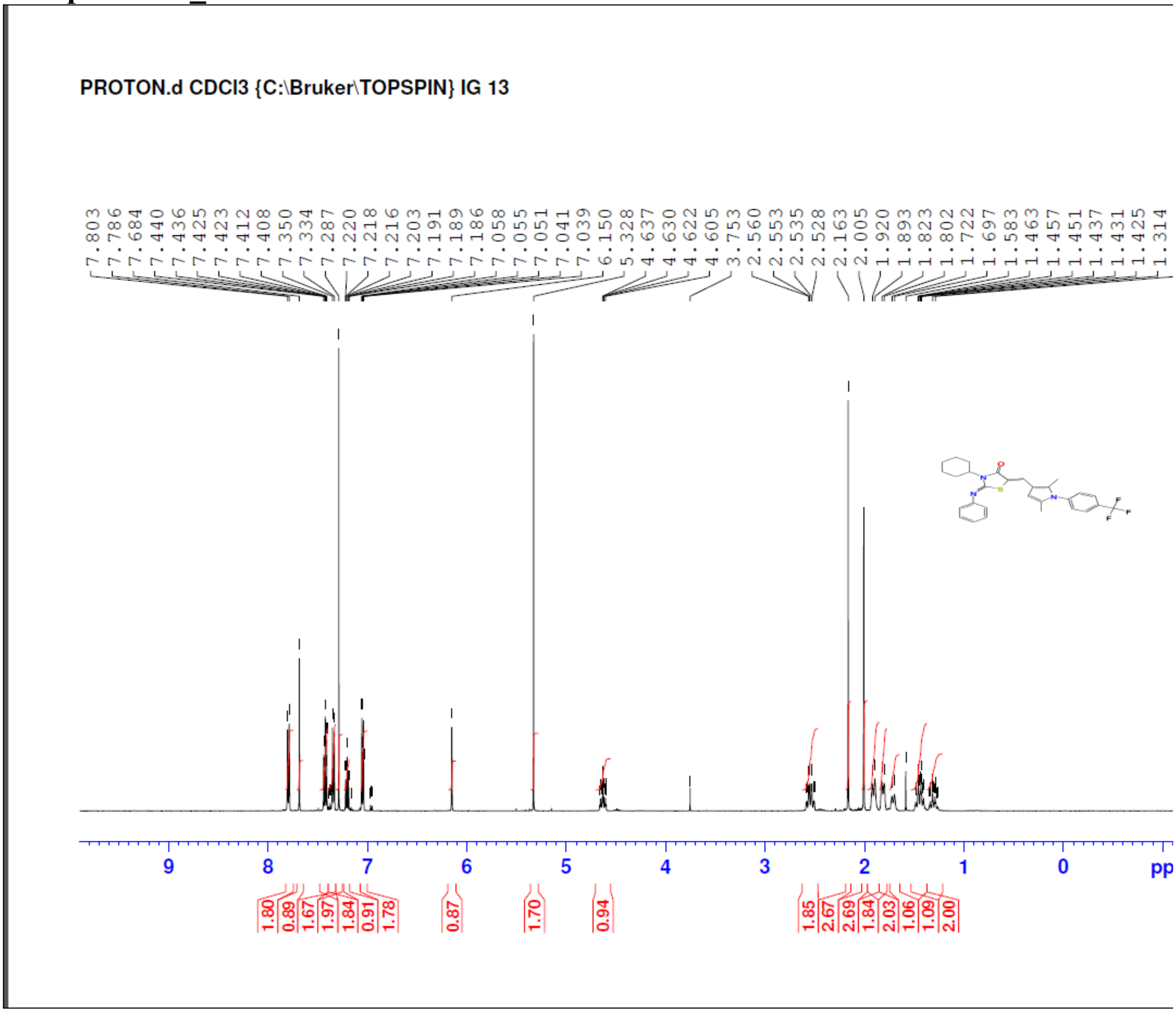

Compound 23_C-NMR

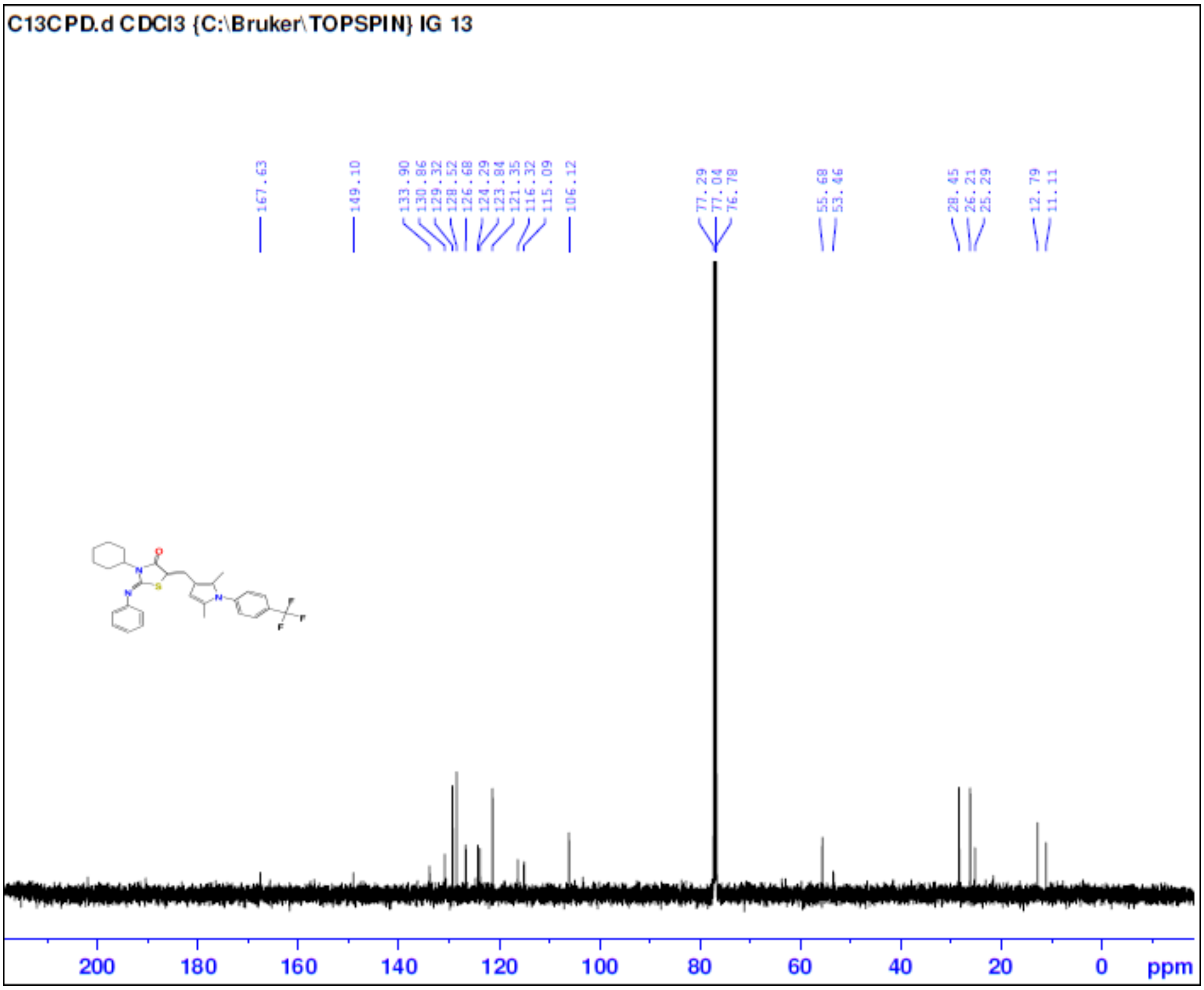

Compound 24_H-NMR

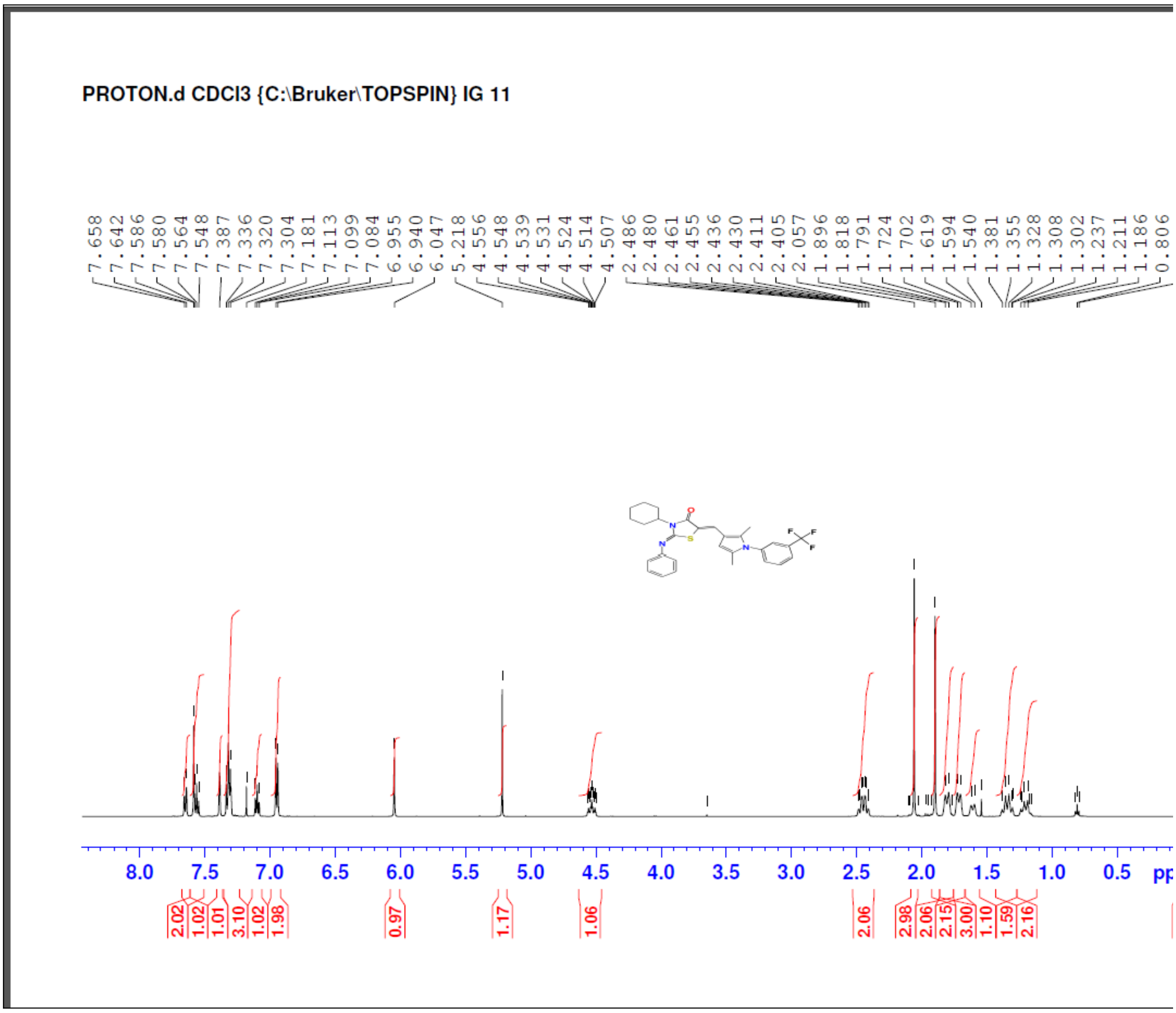

Compound 24_C-NMR

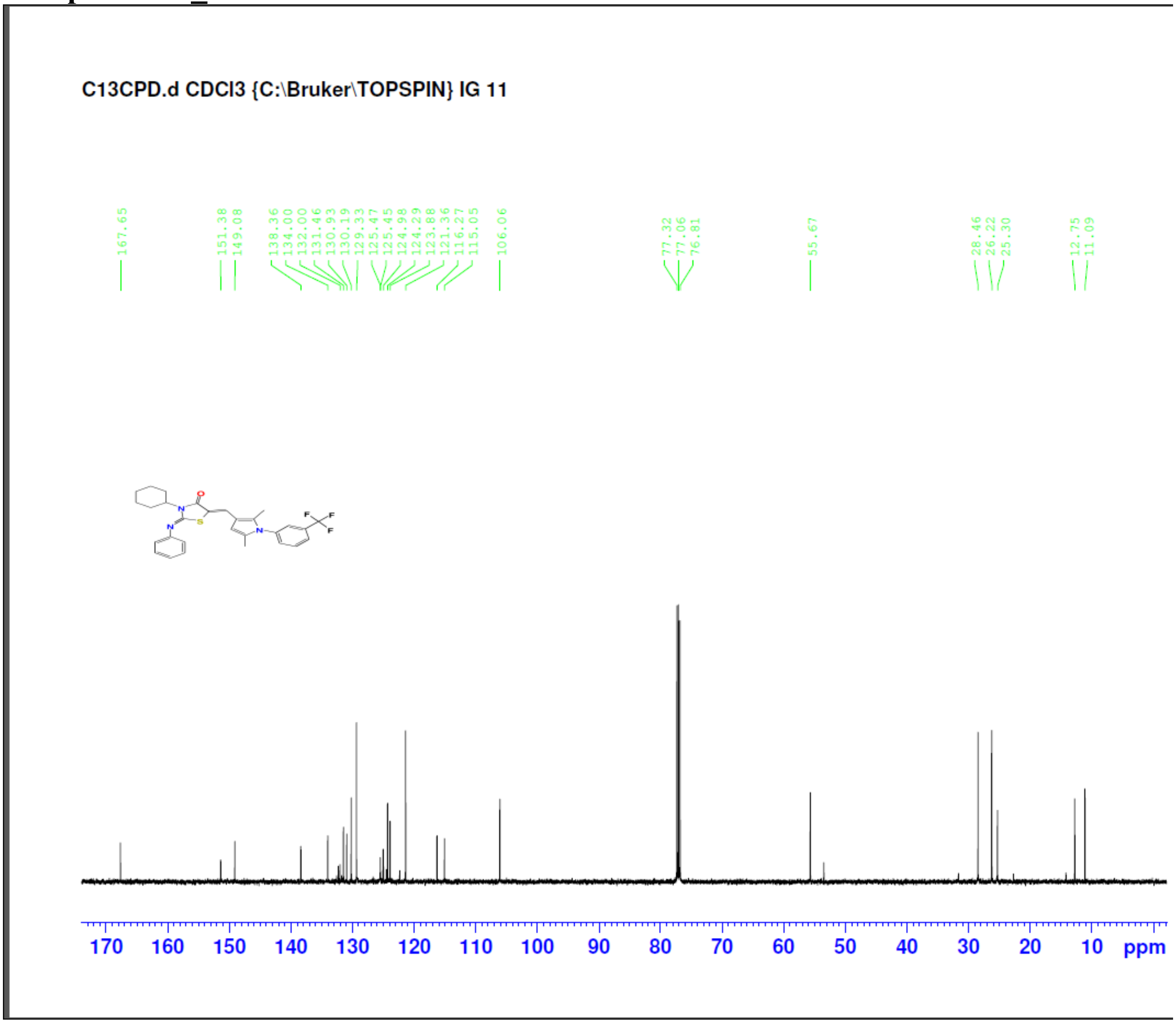

Compound 25_H-NMR

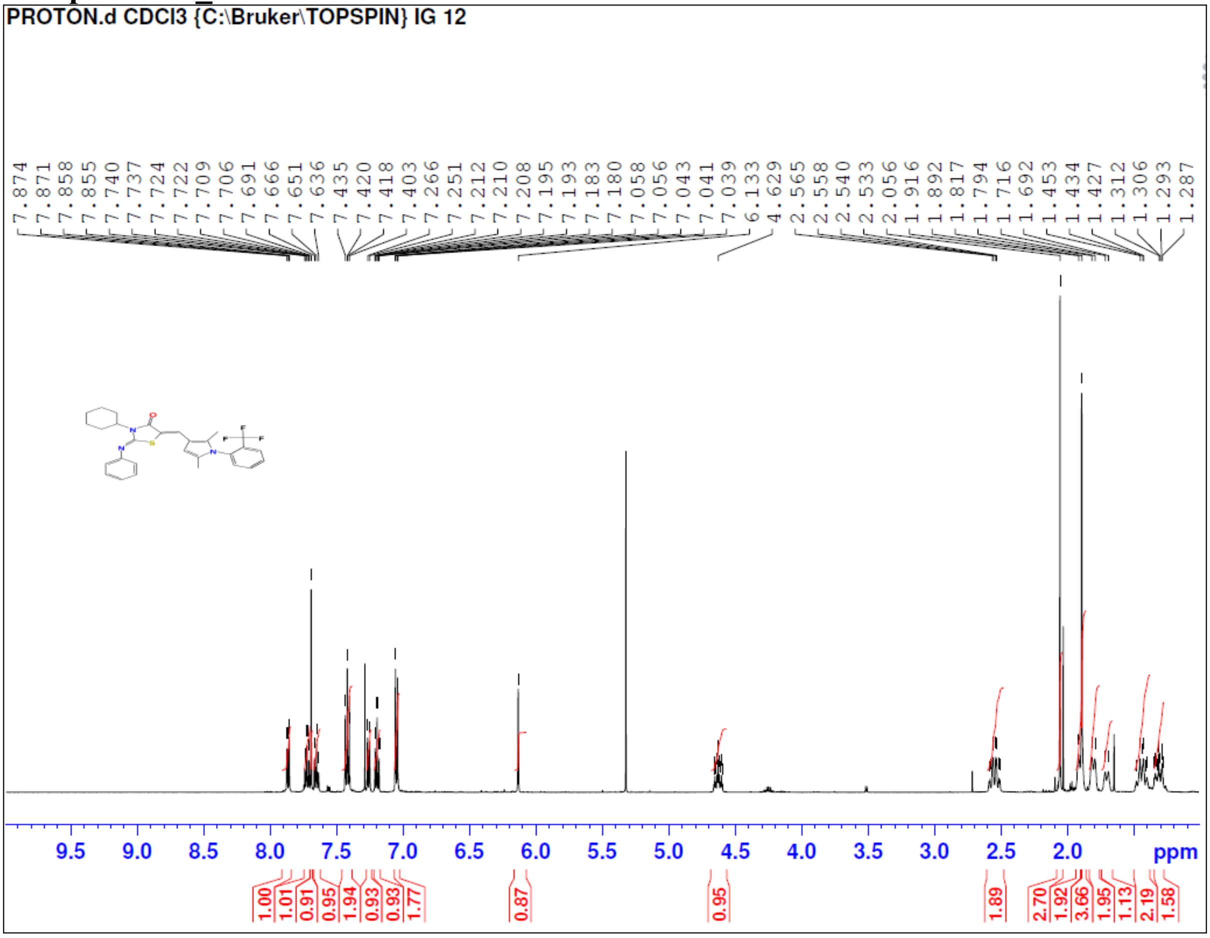

Compound 25_C-NMR

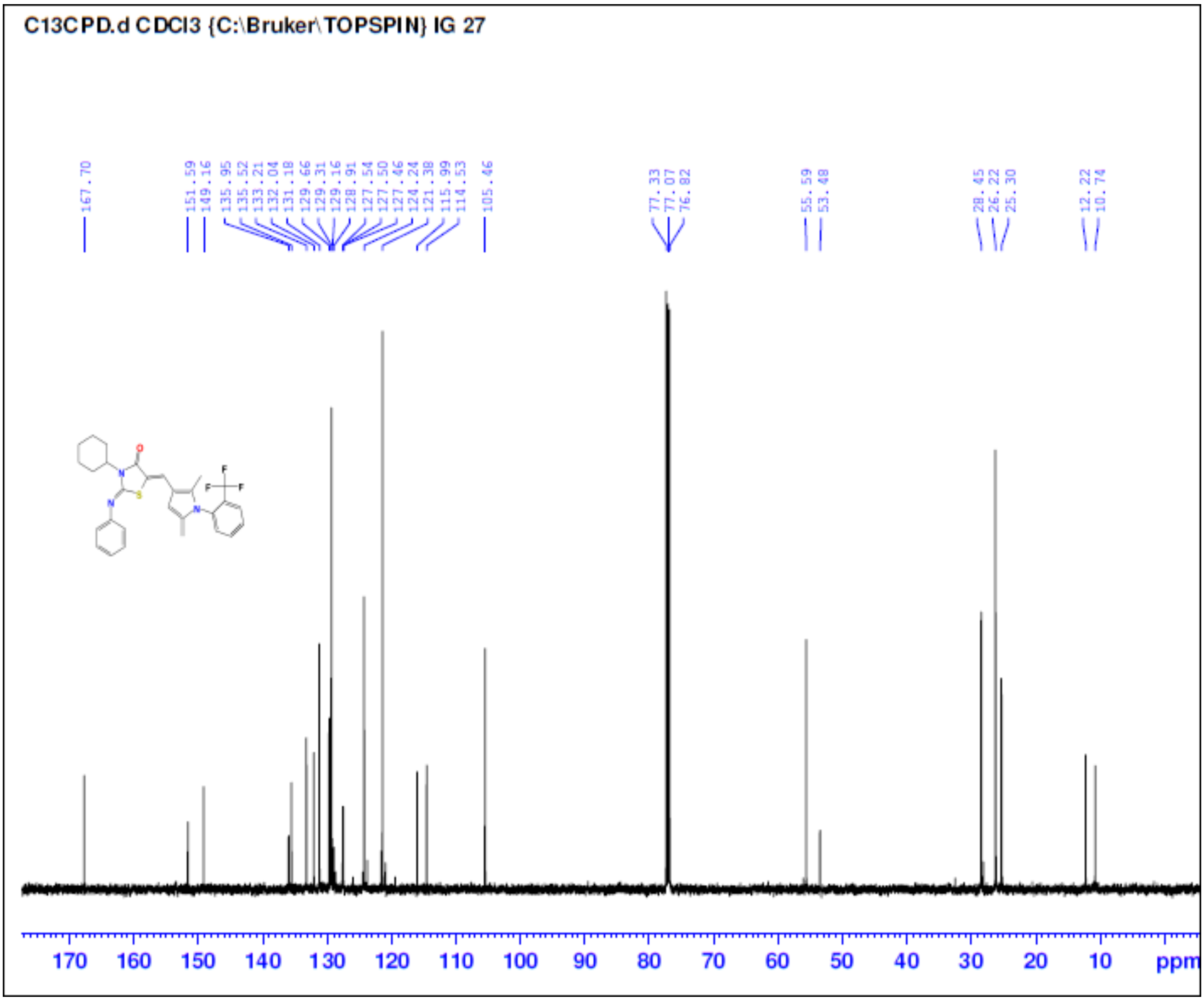

Compound 31_H-NMR

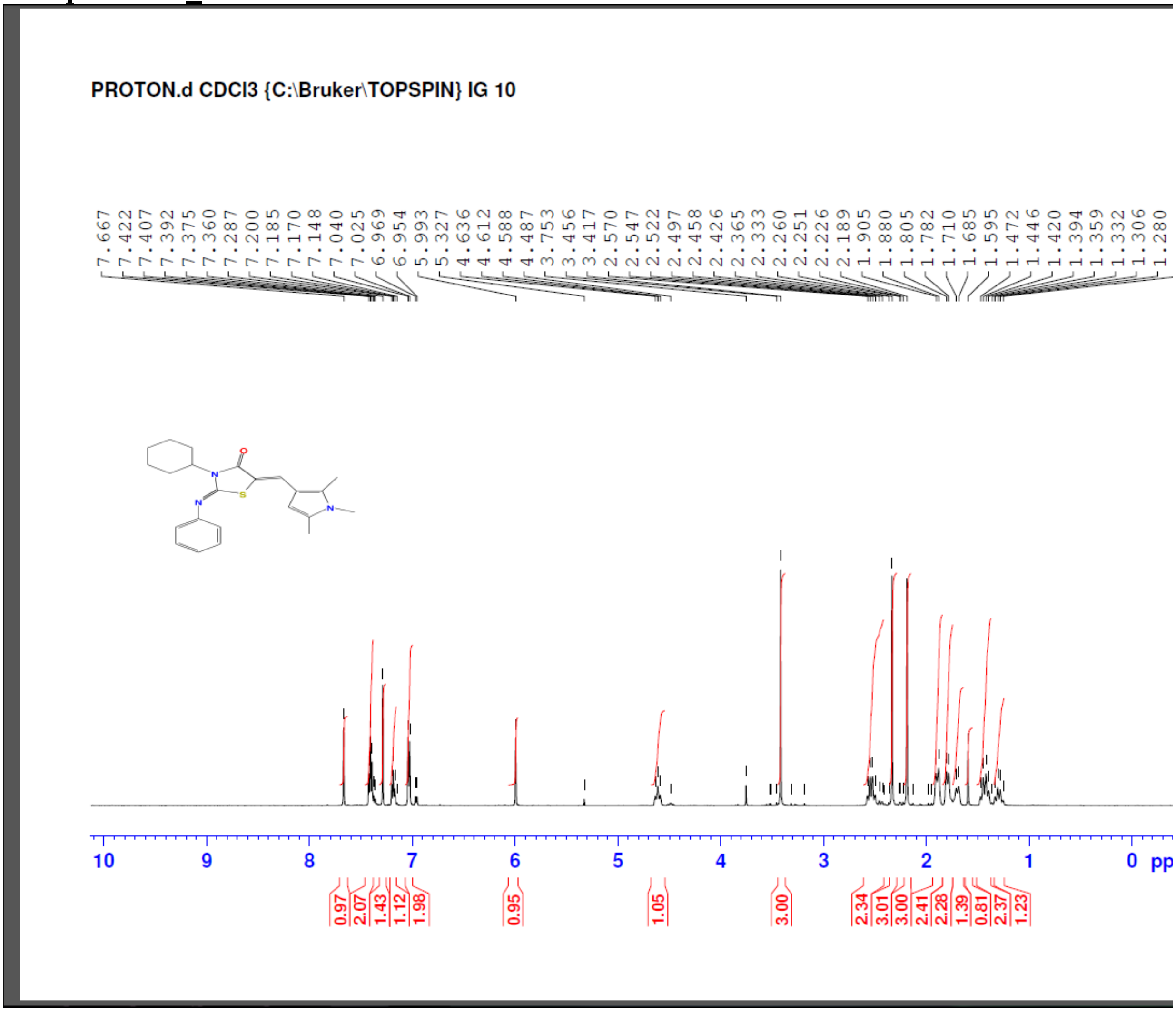

Compound 31_C-NMR

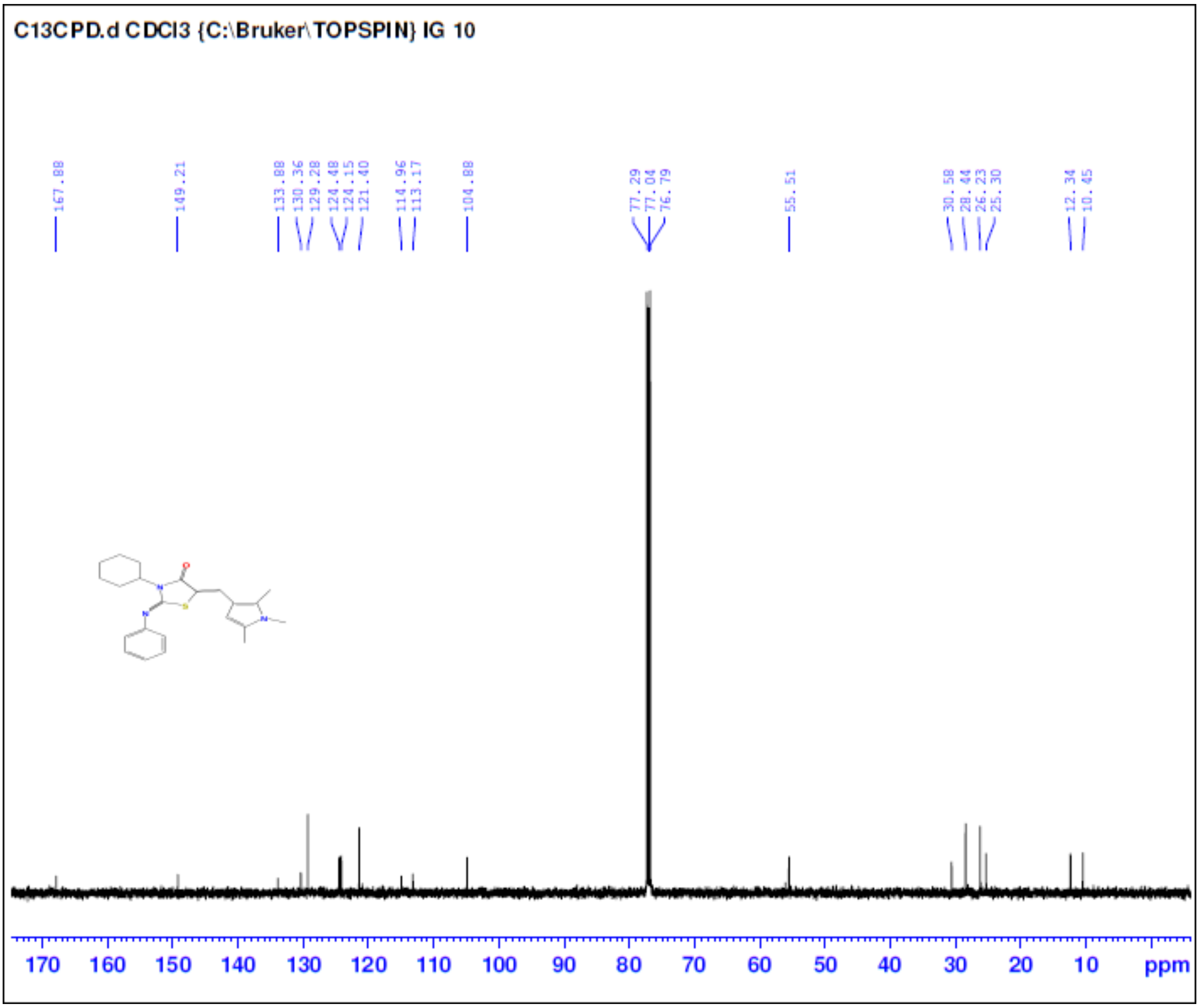

Compound 35_H-NMR

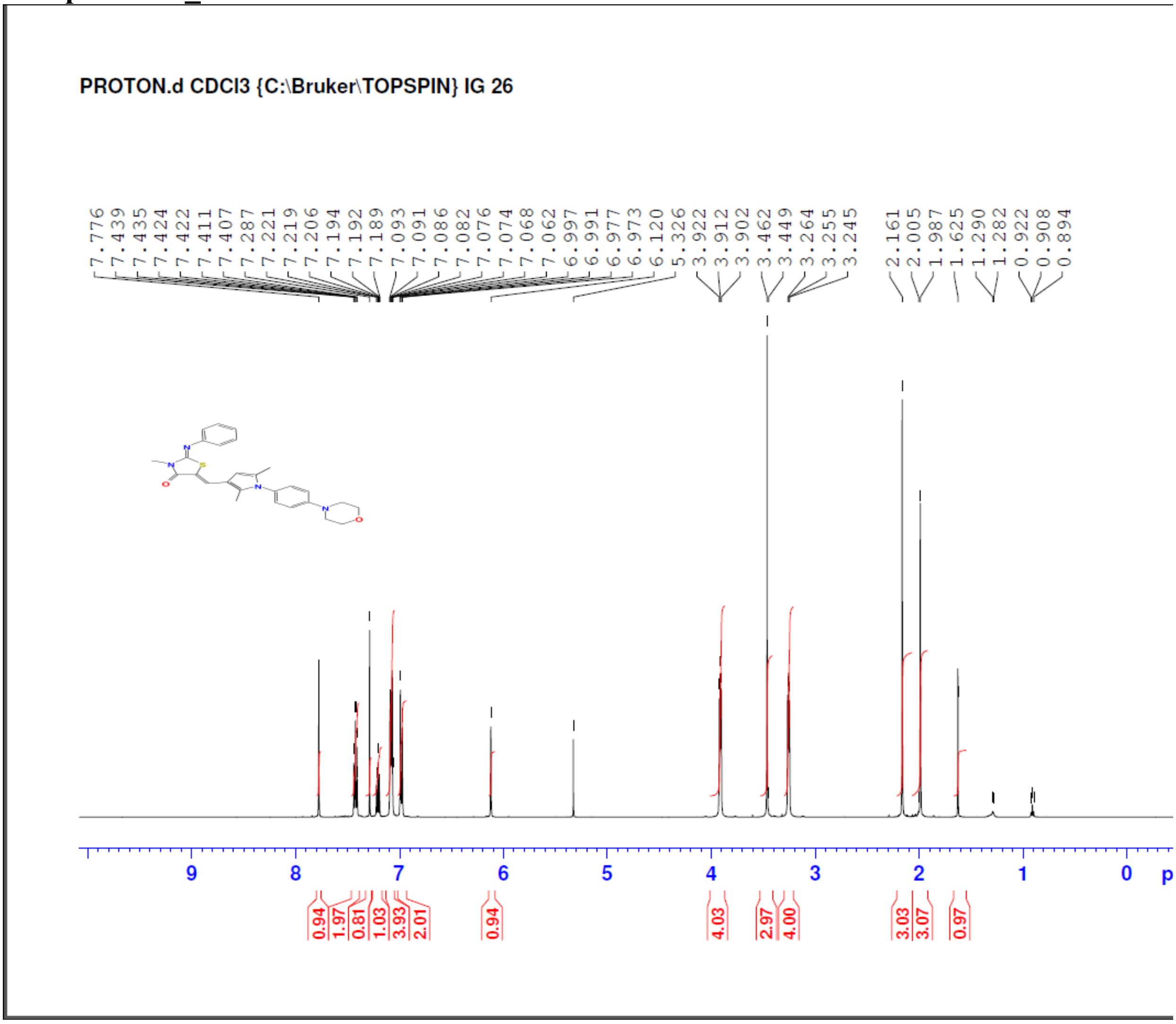

Compound 35_C-NMR

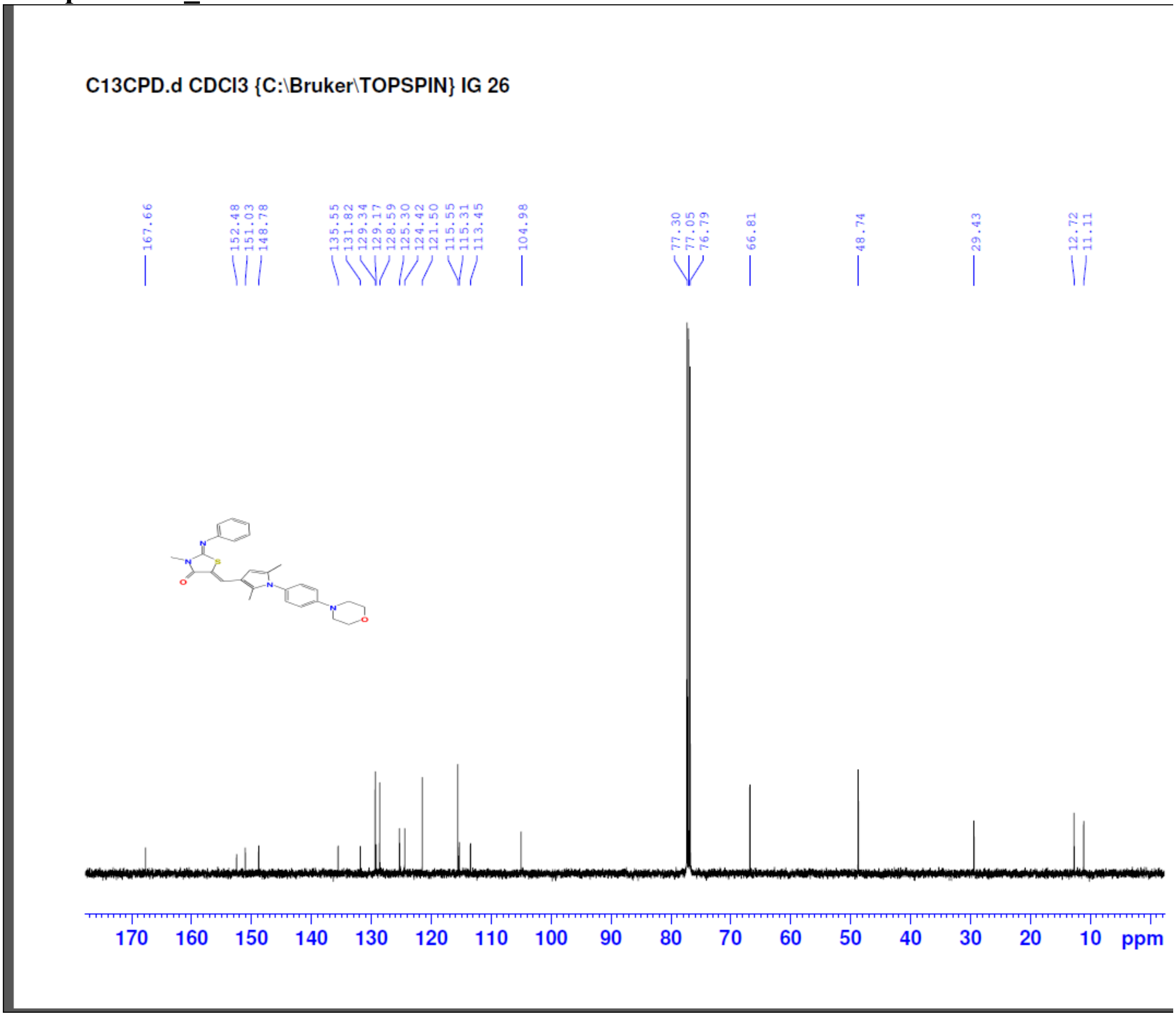

IV. Physicochemical Evaluation

Calculated Parameters: Theoretical physicochemical values were calculated using the ACD/Labs Release 9.0 software (Advanced Chemistry Development, Toronto).

Solubility Estimation: Aqueous solubility was estimated by nephelometry. A concentrated stock solution prepared in DMSO was spiked into either pH 6.5 phosphate buffer or 0.01 N HCl (approx. pH 2.0) with the final DMSO concentration being 1%. Samples were then analyzed by Nephelometry to determine the solubility range as described previously.[9]

Chromatographic Log D Estimation: The partition coefficient ($gLogD_{pH\ 7.4}$) of **20** was estimated by correlation of its chromatographic retention properties with the retention characteristics of a series of standard compounds with known partition coefficient values. Data were collected using a Waters 2785 HPLC with a Waters 2487 dual channel UV detector with a Phenomenex Synergi Hydro-RP 4 μm (30 mm x 2 mm) column. The mobile phase was aqueous buffer (50 mM ammonium acetate, pH 7.4) and acetonitrile with an acetonitrile gradient of 0-100% over 10 min. Compound elution was monitored at 220 and 254 nm.

V. *In Vitro* Metabolism

Compound **20** (1 µM) was incubated at 37 °C with human or mouse liver microsomes (BD Gentest, Discovery Labware Inc., Woburn, MA). The reaction was initiated by the addition of an NADPH-regenerating system containing 1 mg/mL NADP, 1 mg/mL glucose-6-phosphate, 1 U/mL glucose-6-phosphate dehydrogenase, and 0.67 mg/mL $MgCl_2$and quenched at various time points over the incubation period (60 min) by the addition of ice cold acetonitrile. Samples were also incubated in the absence of co-factor to monitor for non-specific degradation. The relative loss of parent compound and formation of metabolic products were monitored by LC/MS using a Waters/Micromass LCT instrument.

Compound concentration versus time data were fitted to an exponential decay function to determine the first-order rate constant for substrate depletion which was used to obtain the in vitro intrinsic clearance (in vitro CL_{int}), and then scaled to predict the human intrinsic clearance (scaling parameters included liver mass of 25.7 g liver/kg body weight for human and 87.5 g liver/kg body weight for mouse and microsomal protein content of 45 mg microsomal protein/g liver mass for both species)[10]. The predicted human blood clearance and hepatic extraction ratio (EH) were then

obtained using the well-stirred model of hepatic clearance with hepatic blood flow rates of 20.7 mL/min/kg (human) and 90.0 mL/min/kg (mouse).

VI. Design and Results of the *in vitro* & *in vivo* Studies.

A. Drug inhibition of *in vitro* cultured *P. falciparum* parasite:[2] All *in vitro* assays were carried out twice independently in duplicate.*In vitro* activity against theerythrocytic stages of *P. falciparum* was determined by using a ^{3}H-hypoxanthine incorporation assay, using the chloroquine and pyrimethamine resistant K1 strain and the standard drugs chloroquine (Sigma C6628) and artemisinin (Arteannuin, Qinghaosu; Sigma 36,159- 3). Compounds were dissolved in DMSO at 10 mg/mL and added to parasite cultures incubated in RPMI 1640 medium without hypoxanthine, supplemented with HEPES (5.94 g/L), $NaHCO_3$ (2.1 g/L), neomycin (100U/mL), Albumax^R (5 g/L), and washed human red cells A+ at 2.5% haematocrit (0.3% parasitaemia). Serial doubling dilutions of each drug were prepared in 96-well microtiter plates and incubated in a humidified atmosphere at 37 °C; 4% CO_2, 3% O_2, 93% N_2. After 48 h, 50 μLof ^{3}H-hypoxanthine (= 0.5 μCi) was added to each well of the plate. The plates were incubated for an additional 24 h under the same conditions. The plates were then harvested with a Betaplate cell harvester (Wallac, Zurich, Switzerland), and the red blood cells were transferred onto a glass fiber filter and then washed with distilled water. The dried filters were inserted into a plastic foil with 10 mL of scintillation fluid and were counted in a Betaplate™ liquid scintillation counter (Wallac, Zurich, Switzerland); IC_{50}values were calculated from sigmoidal inhibition curves using Microsoft Excel.

B. *In vitro* cytotoxicity assay: [2] Assays were performed in 96-well microtiter plates, each well containing 100 μl of RPMI 1640 medium supplemented with 1% L-glutamine (200mM) and 10% fetal bovine serum, and 4 x 10^4 L-6 cells (a primary cell line derived from rat skeletal myoblasts). Serial drug dilutions of seven 3-fold dilution steps covering a range from 90 to 0.123 μg/mL were prepared. After 72 h of incubation the plates were inspected under an inverted microscope to assure growth of the controls and sterile conditions. 10μL of Alamar Blue was then added to each well and the plates incubated for another 2 h. Then the plates were read with a Spectramax Gemini XS microplatefluorometer (Molecular Devices Cooperation, Sunnyvale, CA, USA) using an excitation wave length of 536 nm and an emission wave length of 588 nm. Data were analysed using the microplate reader software Softmax Pro (Molecular Devices Cooperation, Sunnyvale, CA, USA).

C. *In vivo* antimalarial efficacy studies (4 days treatment).[2]

In vivo antimalarial activity was assessed basically as previously described. Groups of three female NMRI mice (20–22 g) intravenously infected with 2 x 10^7 parasitized erythrocytes on day 0 with GFP-transfected *P. berghei* strain ANKA. .Unless otherwise indicated compounds were formulated in 100% DMSO, diluted 10-fold in distilled water and administered intraperitoneally in a volume of 10 mL kg^{-1} on four consecutive days (4, 24, 48 and 72 h post infection). Parasitemia was determined on day 4 post infection (24 h after last treatment) by FACS analysis. Activity was calculated as the difference between the mean per cent parasitaemia for the control (n=5 mice) and treated groups expressed as a per cent relative to the control group. The survival time in days was also recorded up to 30 days after infection. A compound was considered curative if the animal survived to day 30 after infection with no detectable parasites. In vivo efficacy studies in mice were conducted at the Swiss Tropical and Public Health Institute (Basel) according to the rules and regulations for the protection of animal rights ("Tierschutzverordnung") of the Swiss "BundesamtfürVeterinärwesen". They were approved by the veterinary office of Canton Basel-Stadt, Switzerland.

VII. Additional References.

[1] S.M. Ramsh, G.S. Antonova, A.I. Ginak, E.G. Sochilin, Reactivity and tautomerism of azolidines .15. Synthesis of 2-phenylimino-5-arylidenethiazolidin-4-ones and their methylated analogs, Zhurnal Org. Khimii, 11 (1975) 1755-1759.

[2] D. Murugesan, A. Mital, M. Kaiser, D.M. Shackleford, J. Morizzi, K. Katneni, M. Campbell, A. Hudson, S.A. Charman, C. Yeates, I.H. Gilbert, Discovery and Structure-Activity Relationships of Pyrrolone Antimalarials, J. Med. Chem., 56 (2013) 2975-2990.

[3] P.L. Anderson, 1-Phenyl-pyrazole derivatives as glucagon inhibitors, (1982), 4359474

[4] J.A. Ragan, B.P. Jones, M.J. Castaldi, P.D. Hill, T.W. Makowaki, Ullman methoxylation in the presence of a 2,5-dimethykpyrrole-blocked aniline; preparation of 2-fuloro-4-methoxyaniline, Organic Syntheses, 10 (2004) 418.

[5] N.J. Kevin, J.L. Duffy, B.A. Kirk, K.T. Chapman, W.A. Schleif, D.B. Olsen, M. Stahlhut, C.A. Rutkowski, L.C. Kuo, L. Jin, J.H. Lin, E.A. Emini, J.R. Tata, Novel HIV-1 protease inhibitors active against multiple PI-resistant viral strains: coadministration with indinavir, Bioorg. Med. Chem. Lett., 13 (2003) 4027-4030.

[6] G. Sbardella, A. Mai, M. Artico, R. Loddo, M.G. Setzu, P. La Colla, Synthesis and in vitro antimycobacterial activity of novel 3-(1H-pyrrol-1-yl)-2-oxazolidinone analogues of PNU-100480, Bioorg. Med. Chem. Lett., 14 (2004) 1537-1541.

[7] A. Gangjee, J. Patel, R.L. Kisliuk, Y. Gaumont, 5,10-Methylenetetrahydro-5-deazafolic acid and analogues: synthesis and biological activities, J. Med. Chem., 35 (1992) 3678-3685.

[8] R.L. Hinman, S. Theodorou, Methylpyrroles. Synthesis and Characterization, J. Org. Chem., 28 (1963) 3052-3058.

[9] C.D. Bevan, R.S. Lloyd, A high-throughput screening method for the determination of aqueous drug solubility using laser nephelometry in microtiter plates, Anal. Chem., 72 (2000) 1781-1787.

[10] R.S. Obach, Prediction of human clearance of twenty-nine drugs from hepatic microsomal intrinsic clearance data: An examination of in vitro half-life approach and nonspecific binding to microsomes, Drug Metab. Dispos., 27 (1999) 1350-1359.
